# Hotspots of uncertainty in land‐use and land‐cover change projections: a global‐scale model comparison

**DOI:** 10.1111/gcb.13337

**Published:** 2016-06-08

**Authors:** Reinhard Prestele, Peter Alexander, Mark D. A. Rounsevell, Almut Arneth, Katherine Calvin, Jonathan Doelman, David A. Eitelberg, Kerstin Engström, Shinichiro Fujimori, Tomoko Hasegawa, Petr Havlik, Florian Humpenöder, Atul K. Jain, Tamás Krisztin, Page Kyle, Prasanth Meiyappan, Alexander Popp, Ronald D. Sands, Rüdiger Schaldach, Jan Schüngel, Elke Stehfest, Andrzej Tabeau, Hans Van Meijl, Jasper Van Vliet, Peter H. Verburg

**Affiliations:** ^1^ Environmental Geography GroupDepartment of Earth SciencesVrije Universiteit AmsterdamDe Boelelaan 10871081 HVAmsterdamThe Netherlands; ^2^ School of GeoSciencesUniversity of EdinburghDrummond StreetEdinburghEH89XPUK; ^3^ Department Atmospheric Environmental Research (IMK‐IFU)Karlsruhe Institute of TechnologyKreuzeckbahnstr. 1982467Garmisch‐PartenkirchenGermany; ^4^ Joint Global Change Research InstitutePacific Northwest National LaboratoryCollege ParkMD20740USA; ^5^ PBL Netherlands Environmental Assessment AgencyP.O. Box 3033720AH BilthovenThe Netherlands; ^6^ Department of Geography and Ecosystem ScienceLund UniversitySölvegatan 12LundSweden; ^7^ Center for Social and Environmental Systems ResearchNational Institute for Environmental Studies16‐2 OnogawaTsukubaIbaraki305‐8506Japan; ^8^ Ecosystem Services and Management ProgramInternational Institute for Applied Systems AnalysisA‐2361LaxenburgAustria; ^9^ Potsdam Institute for Climate Impact Research (PIK)P.O. Box 60 12 0314412PotsdamGermany; ^10^ Department of Atmospheric SciencesUniversity of IllinoisUrbanaIL61801USA; ^11^ Resource and Rural Economics DivisionEconomic Research ServiceUS Department of AgricultureWashingtonDC20250USA; ^12^ Center for Environmental Systems ResearchUniversity of KasselWilhelmshöher Allee 47D‐34109KasselGermany; ^13^ LEIWageningen University and Research CentreP.O. Box 297032502LS The HagueThe Netherlands; ^14^ Swiss Federal Research Institute WSLZürcherstrasse 111CH‐8903BirmensdorfSwitzerland

**Keywords:** land‐use allocation, land‐use change, land‐use model uncertainty, map comparison, model intercomparison, model variation

## Abstract

Model‐based global projections of future land‐use and land‐cover (LULC) change are frequently used in environmental assessments to study the impact of LULC change on environmental services and to provide decision support for policy. These projections are characterized by a high uncertainty in terms of quantity and allocation of projected changes, which can severely impact the results of environmental assessments. In this study, we identify hotspots of uncertainty, based on 43 simulations from 11 global‐scale LULC change models representing a wide range of assumptions of future biophysical and socioeconomic conditions. We attribute components of uncertainty to input data, model structure, scenario storyline and a residual term, based on a regression analysis and analysis of variance. From this diverse set of models and scenarios, we find that the uncertainty varies, depending on the region and the LULC type under consideration. Hotspots of uncertainty appear mainly at the edges of globally important biomes (e.g., boreal and tropical forests). Our results indicate that an important source of uncertainty in forest and pasture areas originates from different input data applied in the models. Cropland, in contrast, is more consistent among the starting conditions, while variation in the projections gradually increases over time due to diverse scenario assumptions and different modeling approaches. Comparisons at the grid cell level indicate that disagreement is mainly related to LULC type definitions and the individual model allocation schemes. We conclude that improving the quality and consistency of observational data utilized in the modeling process and improving the allocation mechanisms of LULC change models remain important challenges. Current LULC representation in environmental assessments might miss the uncertainty arising from the diversity of LULC change modeling approaches, and many studies ignore the uncertainty in LULC projections in assessments of LULC change impacts on climate, water resources or biodiversity.

## Introduction

Land‐use and land‐cover (LULC) change has been identified as a major driver of global and regional environmental change and is increasingly recognized in today's assessment of anthropogenic impacts on the environment on a global scale (Foley *et al*., 2005; Brovkin *et al*., 2013; Verburg *et al*., 2015). While natural forces dominated the appearance of the land's surface for billions of years, humans are now recognized as the main driver shaping the environment in the modern world (Ellis, 2011). Agricultural activity, forest management and the demand for energy have increasing impacts on the functioning of the Earth system.

Human‐induced LULC changes are estimated to contribute substantially to anthropogenic emissions of CO_2_ (Houghton *et al*., 2012; Le Quere *et al*., 2015) and non‐CO_2_ greenhouse gases (GHG) to the atmosphere (Smith *et al*., 2014; Tubiello *et al*., 2015). GHG emissions related to LULC change, however, represent the biggest source of uncertainty in the global carbon budget (Ballantyne *et al*., 2015). Beyond biogeochemical impacts on the carbon and nitrogen cycles, LULC change and land management have been identified to alter biophysical characteristics of the earth's surface (e.g., albedo, soil moisture and surface roughness) especially in regions of intense past LULC change (Pitman *et al*., 2009; De Noblet‐Ducoudre *et al*., 2012). This in turn will have feedbacks to the climate system (Luyssaert *et al*., 2014; Mahmood *et al*., 2014; Rounsevell *et al*., 2014).

To assess the direction and strength of anthropogenic LULC change effects on ecosystems and the climate, environmental assessments heavily rely on the provision of historical reconstructions and future projections of LULC change trajectories generated by models. Thus, the estimates are also affected by uncertainties originating in the underlying model data on anthropogenic LULC change for historical and future times (Meiyappan & Jain, 2012; Klein Goldewijk & Verburg, 2013). Future LULC change information is usually provided by either integrated assessment models (IAMs) or specialized land‐use models (LUMs) to downstream models such as Earth system models (ESMs), global vegetation models (DGVMs) or other ecosystem model applications. While the uncertainty in the reconstruction of historic LULC changes has been assigned to different approaches in the reconstruction method and the limited data availability for historic times (Ellis *et al*., 2013; Klein Goldewijk & Verburg, 2013), future model projections suffer from the lack of a validation option and are dependent on the underlying scenario storylines. Large efforts have been made to develop and improve simulations of future LULC on a global scale by different disciplines and modeling approaches (Michetti & Zampieri, 2014; NRC, 2014). However, uncertainties remain and originate from different sources in the LULC change modeling process (Verburg *et al*., 2013).

Global‐scale LULC change models (both IAMs and LUMs) are difficult to evaluate against observational data for historical and recent times due to the lack of suitable global observations and independent datasets, which are not used in model calibration (Verburg *et al*., 2011). Instead of evaluation, model intercomparison exercises have been conducted to obtain insight in the differences in models. While there have been some comparison exercises at regional scale (Busch, 2006; Pontius *et al*., 2008; Mas *et al*., 2014), global‐scale comparisons have been constrained to the larger integrated assessment and macro‐economic models, such as in the Agricultural Model Intercomparison and Improvement Project (AgMIP) (Nelson *et al*., 2014a,b; Schmitz *et al*., 2014), the Inter‐Sectoral Impact Model Intercomparison Project (ISI‐MIP) (Nelson *et al*., 2014a; Warszawski *et al*., 2014) or the EMF27 intercomparison exercise on land use (Popp *et al*., 2014b). These comparisons address several model outcomes, but not the simulated spatial LULC change patterns. Recently, a broader set of modeled LULC change scenarios was compared (P. (Alexander et al., in review)). However, this comparison also focused on the simulated global quantity of LULC change, without differentiating uncertainties to different regions, specific LULC conversions or grid cell locations.

Understanding of spatial patterns of LULC changes is essential, because these spatial patterns affect important biogeochemical, biophysical and ecological variables such as soil fertility, local climate and biodiversity. For example, the climate impact of converting forest into agricultural land might be different from the conversion of grazing land into agricultural land (Guo & Gifford, 2002; Don *et al*., 2011; Mahmood *et al*., 2014). Moreover, the spatial patterns of LULC change identify those locations and people that will face large changes in their environment. Thus, spatially explicit assessment of uncertainties is required to identify not only the amount but also the geographic extent and location of uncertainty.

The main objective of this study was to compare a wide range of existing global‐scale LULC projections in terms of spatial variability and land conversion processes. To reach this objective, the outputs of a set of 11 global‐scale LULC change models (providing LULC projections based on 43 scenarios) are compared on both a regional level and a spatially gridded level. These 43 scenarios represent a diverse range of biophysical and socioeconomic assumptions about the future and capture a broad range of regional‐ and gridded‐level uncertainties typical in current models, therefore allowing to investigate in which regions LULC change projections are least and most uncertain and at which grid cell locations models agree and disagree about future LULC developments.

## Materials and methods

### Models and scenarios

**Figure 1:**
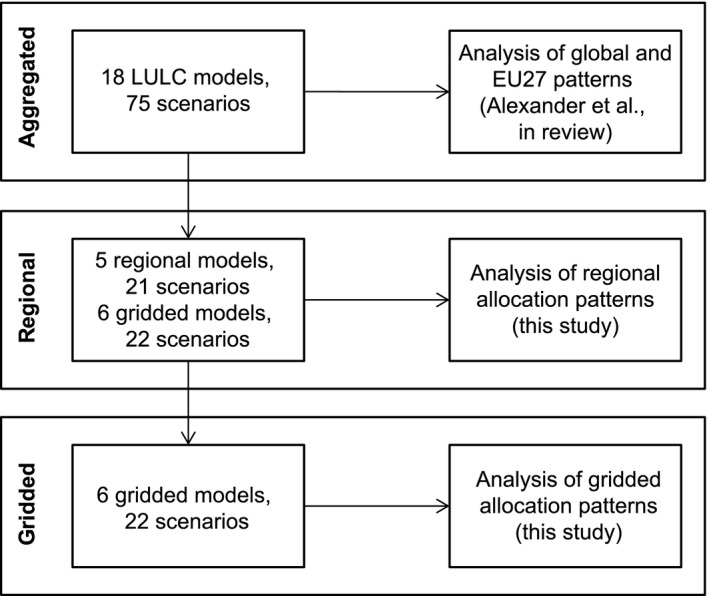
Overview of the LUC4C model intercomparison exercise; global and EU27 quantities were analyzed in a separate study ((Alexander et al., in review), in review) while an adjusted database was used for the regional and spatially gridded analysis in this study.

Our comparison included 11 models covering a total of 43 scenarios (Table 1), which represent a subset of the database collected for the analysis of global and European quantities of LULC change in Alexander et al. (in review). Models which provide only output aggregated at the global level or only cover the European continent were not considered, as they were not suitable for the comparison of regional and gridded spatial patterns of LULC changes in this study. Thus, our comparison is comprised of five models that provide results at world region level and six spatially explicit LULC change models (Fig. 1). To ensure wide participation of models in the intercomparison, modeling teams were invited to submit existing simulations rather than run new simulations with constrained scenario inputs. Most of the scenarios are based on the *shared socioeconomic pathways* (SSP) and *representative concentration pathways* (RCP) framework (Van Vuuren *et al*., 2011; O'Neill *et al*., 2015) or on the previous IPCC *special report on emissions scenarios* (SRES) framework (Nakicenovic & Swart, 2000). However, a few models provided scenarios based on other storylines (Table 1). The LandSHIFT scenarios are based on several biofuel pathways for Germany applying different intensity assumptions for the type of usage (fuel or electricity and heat) and sustainability politics (business‐as‐usual vs. strict environmental regulations). The CLUMondo scenarios on the other hand are driven by demands for crop production, livestock and urban area based on FAO projections (Alexandratos & Bruinsma, 2012). Additional demands for carbon storage and protected areas were used to explore the consequences of different mitigation policies (reduction in GHG emissions and prevention of biodiversity loss) on land change trajectories ((Eitelberg et al., in review)., in review).

**Table 1:** Overview of models and scenarios included in the comparison of regional and gridded land‐use and land‐cover projections. The scenarios based on SSPs are preliminary implementations of the SSP scenarios

Model name	Spatial resolution	LULC types	Temporal resolution	Model type (classification)	Scenario descriptions (number of scenarios)	Key publication(s)
AIM	17 regions	Cropland, Pasture, Forest (managed, unmanaged), Urban, Other Natural	2005, 2010, 2030, 2050 and 2100	CGE	Scenarios based on SSP1, SSP2, SSP3. (3)	Fujimori *et al*. (2012), Hasegawa *et al*. (2014)
FARM	13 regions	Cropland, Pasture, Forest	2010–2050; decadal	CGE	Scenarios based on SSP1, SSP2 and SSP3, each under the current climate and climate scenario RCP 4.5, RCP 6.0 and RCP 8.5, respectively. (6)	Nelson *et al*. (2014a); Sands *et al*. (2014)
GCAM	32 regions	Cropland (irrigated, non‐irrigated, permanent), Pasture (intensive, extensive), Forest (managed, unmanaged), Urban, Other Natural (vegetated, unvegetated)	2010–2100; decadal	PE	Scenarios based on SSP1, SSP2, SSP3, SSP4 and SSP5. (5)	Calvin *et al*. (2013)
MAGNET	26 regions	Cropland, Pasture	2007, 2010, 2020, 2030, 2050 and 2100	CGE	Scenarios based on SSP1, SSP2 and SSP3. (3)	Van Meijl *et al*. (2006), Woltjer *et al*. (2014)
PLUM	157 countries	Cropland, Pasture, Forest	1990–2100; annual	Rule‐based	SRES A1, A2, B1 and B2. (4)	Engström *et al*. (2016)
CAPS	0.5 × 0.5 degree	Cropland, Pasture	2005, 2030, 2050 and 2100	Hybrid^a^	Scenarios based on SSP3, SSP5, RCP 4.5 and RCP 8.5, each under estimated model parameters from historical data from Ramankutty *et al*. (2008) and HYDE (Klein Goldewijk *et al*., 2011). (8)	Meiyappan *et al*. (2014)
CLUMondo	9.25 × 9.25 km grid	Cropland, Pasture, Forest, Urban, Other Natural	2000–2040; annual	Hybrid^a^	FAO 4Demand, Carbon, Potential Protected Area. (3)	Van Asselen & Verburg (2013); Eitelberg et al. (in review) (in review)
GLOBIOM	5 × 5 arcminute grid	Cropland, Pasture, Forest, Other Natural	2010–2100; decadal	PE	Scenarios based on SSP1, SSP2, SSP3. (3)	Havlik *et al*. (2014)
IMAGE	0.5 × 0.5 degree grid	Cropland, Pasture, Forest, Urban, Other Natural	1700–2100; annual	Hybrid^a^	Scenarios based on SSP2 reference and high bioenergy demand scenario under RCP 2.6. (2)	Stehfest *et al*. (2014)
LandSHIFT	5 × 5 arcminute grid	Extended GlobCover legend	2005–2050; 5‐year steps	Rule‐based	Fuel and heat scenarios, with both BAU and regulation assumptions. (4)	Schaldach *et al*. (2011)
MAgPIE	0.5 × 0.5 degree grid	Cropland (irrigated, non‐irrigated), Pasture, Forest, Urban, Other Natural	1995–2100; 5‐year steps	PE	Scenarios based on SSP2 BAU and bioenergy and CCS. (2)	Lotze‐Campen *et al*. (2008), Popp *et al*. (2014a)

a Hybrid models use demand from CGE or PE and allocate to particular grid cells.

Despite these similarities in the underlying scenario framework, models have been applied for a diverse range of biophysical and socioeconomic scenario inputs. For example, some scenarios originate from studies comparing climate mitigation options to business‐as‐usual conditions within the same general storyline (e.g., IMAGE and MAgPIE), while others represent the different SSP storylines considering different historic LULC change or future climate change trajectories (e.g., FARM, CAPS). Further, some of the scenarios include climate impacts on the land sector, while others assume constant climate conditions or use the climatic outcomes in the scenarios as emissions mitigation targets. While often uncertainty in LULC projections is represented by differences between scenarios, the different ways of implementing the same scenario may also lead to different outcomes. Rather than forcing all models to simulate the same scenario, as is done in earlier model comparisons (Schmitz *et al*., 2014), our approach allows us to address the wider range of uncertainties involved in LULC change projections and compare the variation in outcomes as result of different scenarios to the variation resulting from other sources of uncertainty.

### Data preprocessing

Due to this wide range of model and scenario inputs, which were not harmonized prior to the simulations, the model outputs used in our comparison required several steps of preprocessing to allow a meaningful comparison.

For the regional‐level comparison, 12 common world regions were defined by aggregating areas for cropland, pasture and forest (Table S1, Fig. S1). Most of the spatial aggregation, which was necessary due to the variety of regional subdivisions (Table 1), could be achieved by simply adding the areas of two or more regions. In cases, where this was not possible, we rescaled the modeled areas based on the areas reported by FAO country‐level statistics in 2010 (FAOSTAT, 2015) (Table S2). Gridded model results were also included in the regional‐level comparison by simple aggregation of the pixel‐based results to the world regions. As only a small number of the models provided additional land‐use and land management categories (e.g., urban or managed forest), these categories were excluded from the regional part of the analysis. The models start their simulations in different years (Table 1) and report high variation in initial areas for individual LULC types due to differences in category definitions and uncertainty in land statistics (Verburg *et al*., 2011). To adjust for this discrepancy, the modeled absolute area of each LULC type in year 2010 was used as a reference and changes were calculated for the remaining years as proportion of the areas in 2010.

For the gridded‐level comparison, the maps were harmonized to fractions of the grid cell area at a 0.5 x 0.5 degree grid (unprojected WGS84 coordinate system). This ensured the lowest impact on original model outputs and could be achieved by spatial aggregation for CLUMondo, GLOBIOM and LandSHIFT. CAPS, IMAGE and MAgPIE output maps were already provided at the target resolution. The thematic resolution varied widely between the gridded models. For example, the CAPS model only reports cropland and pasture, while the LandSHIFT legend is based on the GlobCover classification, comprising of 30 different LULC types (Bontemps *et al*., 2011). To resolve this thematic diversity, we aggregated all legends to a common legend of cropland, pasture, forest, urban and other natural, as these classes were reported by a majority of the models. When classes were missing, they were assumed to be merged with the other natural category. Details on how individual model outputs have been preprocessed prior to the analysis are reported in the SI.

### Comparison metrics

Different comparison metrics were applied to the regional and spatially explicit model results (Fig. S2). First, coefficients of variation (standard deviation divided by mean, COV) were calculated for each of the 12 world regions based on all scenarios for both the LULC changes (relative to 2010 areas) and LULC areas (areas actually reported in a certain year) at every decadal end year (2010–2100). This allowed to depict variation across the model results with and without the effect of differences in the starting conditions. The coefficient of variation was chosen to provide a comparable measure to describe the spatial pattern of variability across regions. Additionally, median values of LULC changes were used to identify direction and amount of overall LULC change projected by the scenario set.

To assess the sources of uncertainty across LULC types and regions, a regression analysis and analysis of variance (anova) were conducted. We thereby followed Alexander et al., (in review), who ran linear multiple regressions for each LULC type and decadal end year to identify significant drivers of variation in the data. Every scenario in our database was parameterized according to nine common variables that characterize the model, the scenario and the initial condition delta (Table 2, Table S5). This set of explanatory variables was derived by the authors and selected to sufficiently depict the most important differences across the diversity of models and scenarios in our analysis. Results from analysis of robustness tests conducted in Alexander et al., (in review) suggest that upon including alternative variables, no substantially different results are obtained. The modeled LULC area in a certain year was hypothesized to be a function of these nine variables. The full model (including all nine variables for each LULC type and decadal end year) was reduced by stepwise backward selection using the Akaike information criterion (AIC) to avoid over‐fitting and to balance performance and complexity of the regression models (Burnham & Anderson, 2004). Subsequently, an anova was conducted on the regression results to quantify the contribution of each variable to the total variation in the modeled LULC areas. The variation that could not be explained by these variables was summarized in a residual term. As the initial variation was hypothesized as a major reason for uncertainty in the projections (Alexander et al., in review), regression analysis and anova were applied to the LULC areas reported by the models, which include the differences in the starting conditions.

**Table 2:** Overview of variables used to parameterize the scenarios of each model. Details are explained in the SI (Table S3, Table S5)

Variable	Data type	Association
Initial condition delta	Continuous (deviation of model areas from FAO areas in 2010 (FAOSTAT, 2015)	Initial
Model type	Categorical (CGE, PE, Rule‐based, Hybrid)	Model structure
Number of model cells (log)	Continuous	Model structure
CO_2_ concentration 2100	Continuous	Climate scenario
Population 2100	Continuous	Socioeconomic scenario
GDP growth rate to 2100	Continuous	Socioeconomic scenario
Inequality ratio 2100	Continuous	Socioeconomic scenario
Technology change	Discrete (0 = None, 1 = Slow, 2 = Medium, 3 = Rapid)	Socioeconomic scenario
International trade	Discrete (1 = Constrained, 2 = Moderate, 3 = High)	Socioeconomic scenario

To evaluate the uncertainty of LULC change allocation across the six gridded models and identify areas of disagreement among the models, we calculated gridded maps of total variation across all scenarios. Standard deviations of LULC changes at grid cell level were used as a measure of variation. Subsequently, we adapted a pairwise map comparison approach for the LULC areas at grid cell level. Pontius & Cheuk (2006) propose a cross‐tabulation approach to identify disagreement between any two maps at a particular resolution, while considering simultaneously the complete thematic detail of the legend (details provided in the SI). Each entry of the resulting cross‐tabulation matrix can be interpreted as a fraction of the study area (Table S4), which allows quantifying the area of agreement and disagreement between the maps under consideration. Moreover, areas of disagreement can be attributed to particular LULC types (e.g., one model projects forest, while another projects cropland for the same geographic location and point in time). This disagreement will be referred to as ‘confusion’ between LULC types in the remaining paper.

Applying this approach to any two maps (i.e., all unique model and scenario combinations) of the years 2010, 2030, 2050 and 2100 for the six gridded models at the 0.5 × 0.5 degree resolution and the coarsest possible resolution (i.e., the whole globe is taken as one grid cell), we distinguished disagreement between the maps due to different global quantities per LULC type (quantity disagreement) and disagreement due to different allocation of LULC types on the map (allocation disagreement). These two disagreement components add up to the total disagreement at the original resolution (Pontius & Millones, 2011).

To identify grid cell locations of high confusion between LULC types across models and scenarios and visualize the comprehensive information of up to 253 possible pairwise comparisons at the grid cell level (depending on the year considered), mean values for all matrix entries were calculated and aggregated to confusion categories between the main LULC types in the models (cropland, pasture, forest, other natural and urban).

## Results

### Regional‐level change trends and variation in LULC changes

**Figure 2:**
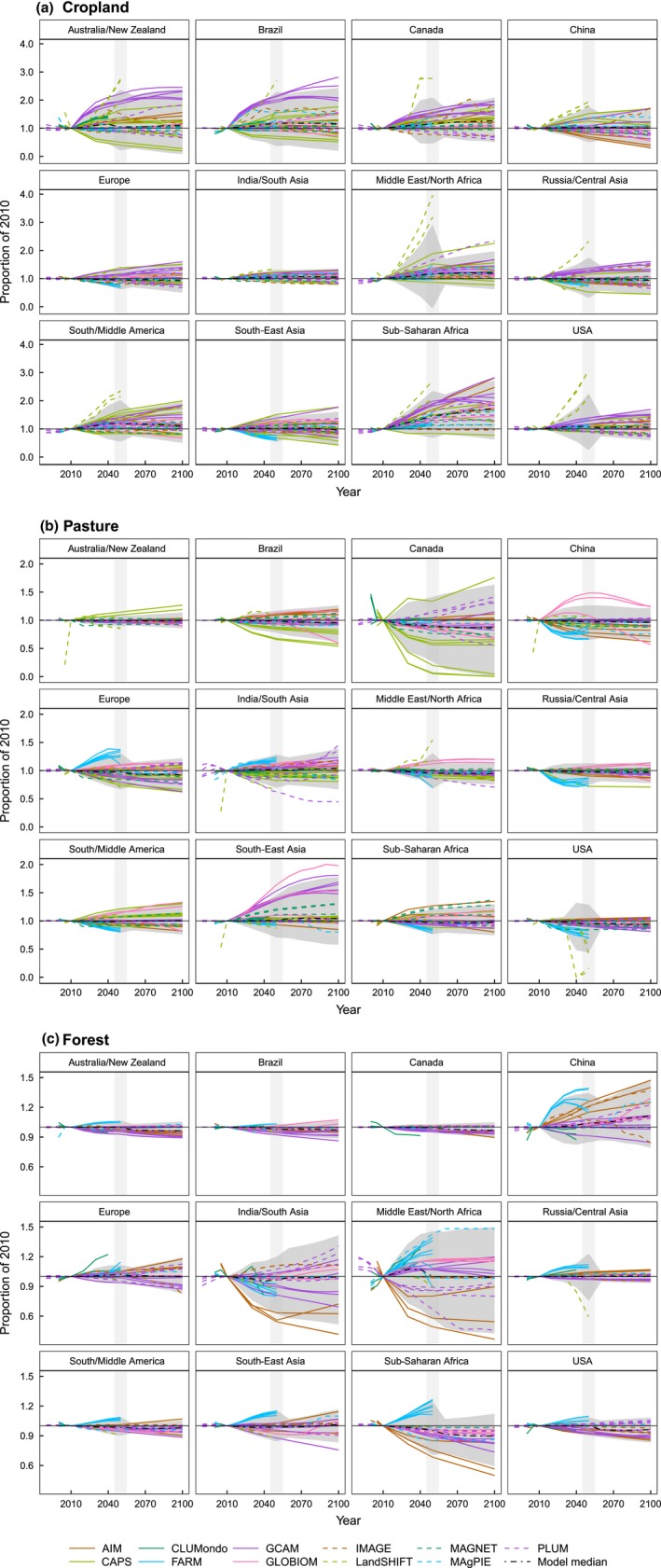
Land‐use and land‐cover change projections for (a) cropland, (b) pasture and (c) forest of 43 scenarios generated by 11 different models. Changes are shown relative to the areas reported in 2010 per category (for original areas projected by the models, see Figure S4). The gray shading represents the 95% interval of model results, while the vertical gray bar indicates a change in the amount of models and scenarios between 2040 and 2060. Note the different ranges of scales applied for cropland, pasture and forest categories.

LULC change projections differ in the direction of change, amount of change and amount of variation among LULC types and regions (Figs 2 and S3). Cropland areas tend to increase in all regions (except for Europe, Russia/Central Asia and South‐East Asia) until the end of the simulation period according to the diverse model and scenario set combined in our study (Table 1). The analysis of median values shows higher rates of cropland expansion in sub‐Saharan Africa (up to 72%), Canada (up to 26%) and Middle East/North Africa (>20%) at the end of the century. In contrast, lower change rates are projected for China (~4% increase) and India/South Asia (~6% increase). Coefficients of variation yielded rather high values in Australia/New Zealand and Brazil (COV > 0.4). In Europe and India/South Asia on the other hand, the models are more in agreement (COV < 0.3). The amount of variation is steadily increasing with time in most of the regions resulting in the highest uncertainty at the end of the simulation period.

Compared with projections of cropland changes, pasture areas show smaller change rates (Fig. 2b). Model median values range between a loss of 13% in Canada and a slight gain of 5% in South‐East Asia in 2100 while in a number of regions hardly any change is shown, for example, in Australia/New Zealand and South/Middle America. The highest variations in pasture change rates are, except for Canada (COV = 0.51), still lower than the lowest COV found in any region for cropland change (COV < 0.3). Australia/New Zealand, Russia/Central Asia and the USA are even below a threshold of 0.1. Except for Canada and South‐East Asia, coefficients of variation show small increase over time.

The forest category shows the lowest overall change rates. However, regions vary for this class in terms of the direction of changes (Fig. 2c). Similar to pasture, some regions show almost no changes in forest areas (e.g., Australia/New Zealand, Brazil, Canada and Europe). Other regions indicate a decrease (sub‐Saharan Africa) or increase (China). In South‐East Asia and India/South Asia, forests are projected to increase in the second half of the century, from a low at around 2050. The highest median values can be found at 10% loss in sub‐Saharan Africa and 11% gain in China at 2100. The level of variation across the wide range of model types and scenarios is rather low for the forest category and smaller than in the pasture category in most regions. The highest COVs are between 0.15 and 0.28 in Middle East/North Africa, India/South Asia, China and sub‐Saharan Africa at the end of the century, while almost all other regions are below a COV of 0.1.

### Regional‐level variation in LULC areas and variance decomposition

**Figure 3:**
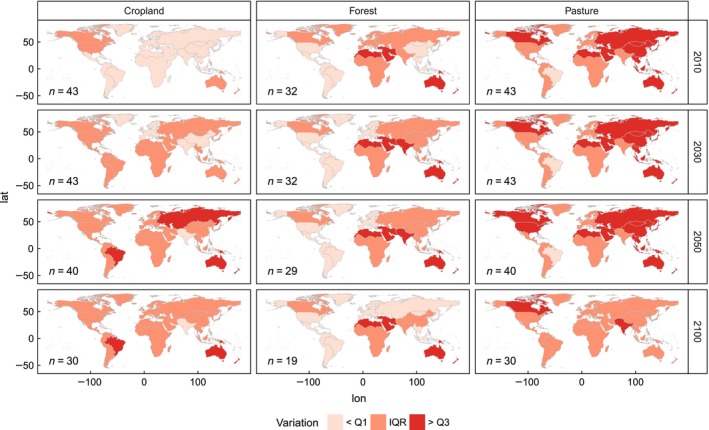
Variation in land‐use areas for 43 scenarios of 11 models in cropland, forest and pasture category; variation expressed as coefficient of variation and classified into lower quartile, interquartile range and upper quartile of the distribution. Quartiles are calculated based on all years and land uses; *n* depicts the number of scenarios underlying the calculation of COV.

Figure 3 shows the COV for each region, calculated based on the areas per LULC type reported by each scenario in 2010, 2030, 2050 and 2100 and classified into lower quartile, interquartile range and upper quartile of the distribution across all LULC types and years. Initial variation in 2010 ranges from a COV of 0.07 for cropland in India/South Asia up to a value of 0.66 for pasture areas in Canada. For cropland only, the highest COVs are in Australia/New Zealand (0.30), the USA (0.21) and Canada (0.20), while the Asian regions, South America, Africa and Europe are lower (0.10–0.20). Pasture has high initial variation (0.21–0.65) in almost every region except for Brazil (0.09). Regional differences in the forest category are smaller, ranging from 0.08 in Middle/South America to 0.43 in Middle East/North Africa and Australia/New Zealand. Despite the regional differences, variation in 2010 areas is generally higher in pasture and forest than in cropland.

A temporal development of coefficients of variation can be seen in the cropland category: in 2030, all regions except for Europe, China and India/South Asia exceed the lower quartile; in 2050, all regions but India/South Asia exceed this threshold; and Australia/New Zealand, Brazil and Russia/Central Asia even turn into the category representing the upper quartile. Cropland projections therefore become more uncertain over time, while hardly any change in variation with time can be detected for pasture and forest.

Although a considerable amount of variation is present already in the 2010 areas for all LULC types, this initial variation is generally larger for forest and pasture than for cropland. Forest and pasture also seem to be more sensitive to changes in our scenario database, as after 2050 (when some of the models end their projections) the amount of variation actually decreases in several regions (e.g., Russia/Central Asia and USA for pasture and Russia/Central Asia and South‐East Asia for forest, respectively).

**Figure 4:**
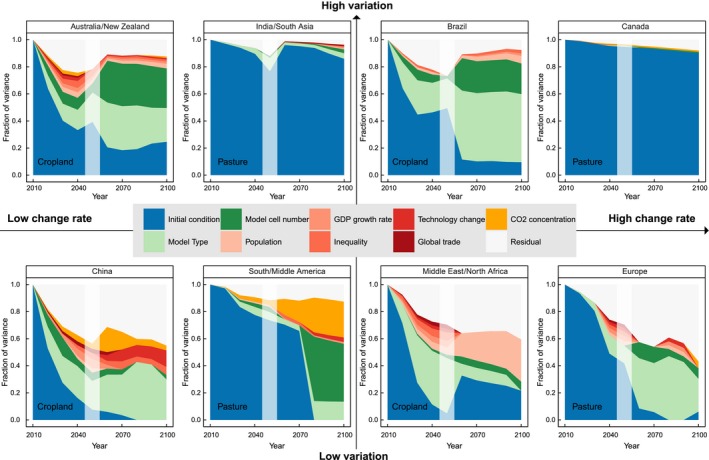
Visualization of variance decomposition for selected regions along the two gradients change rate (horizontal) and variation (vertical). The axes are qualitative based on the distribution of change rates and variation within each LULC type (e.g., Brazil is a representative of high change rates and variations within the cropland category). The order of LULC types within each quadrant is arbitrary. The individual panels show the relative importance of different variance components at each decadal end year. The vertical gray shading indicates a change in the underlying model set between 2040 and 2060.

The dominance of initial uncertainties and the general differences between the LULC types are supported by the variance decomposition (Figs S5–S7). As an example, we show results for selected regions and LULC types in Fig. 4. The contribution of initial conditions in explaining the variation in the scenario results is larger for pasture and forest than for cropland over the whole simulation period and for all regions (except for South/Middle America). Initial conditions explain, for example, almost the total variation in the LULC projections in some regions (India/South Asia and Canada for pasture, Fig. 4). If the initial conditions are not dominating, which is primarily the case for cropland projections, the relative contributions of the remaining explanatory variables are very unevenly distributed across regions. While, for example, in the second half of the simulation period, Australia/New Zealand and Brazil show a high contribution of model parameters for cropland in explaining the variance, scenario parameters contribute almost as much as model parameters in China and Middle East/North Africa for cropland. In Fig. 4, regions are characterized along two gradients: amount of change (i.e., the median value of LULC changes calculated based on all scenarios) and amount of variation (i.e., COV of LULC changes calculated based on all scenarios). The partitioning of variance components shows some general patterns. Generally, the higher the total variation in results, the higher the fraction of variance that can be explained by the initial conditions, which highlights the importance of the base‐year input data in influencing future projections. Although the exact variance fractions are very different across regions, we could not find notable influence of higher overall change rates to the distribution of variance components.

### Gridded‐level variation in LULC changes

**Figure 5:**
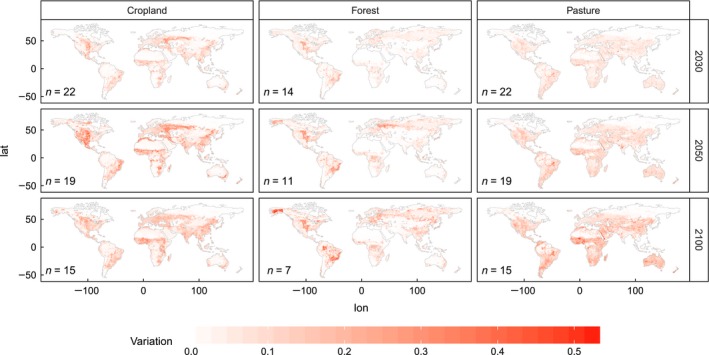
Total variation in net changes (reference year 2010) for cropland, pasture and forest in 2030, 2050 and 2100. The variation is expressed as the standard deviation for each grid cell n depicts the number of scenarios underlying the calculation of standard deviations..

Consistent with the regional‐level results, there is a higher absolute amount of variation in the cropland category than in the forest and pasture categories (indicated by the more intense colors in the cropland maps in Fig. 5).

Hotspots of variation in cropland changes are located in the central United States and north Mexico, the eastern part of Brazil, the boundaries of the Sahara and large parts of western Russia in 2030. Further small areas with high variations appear in the southern part of Africa (Zimbabwe and Madagascar), some parts of India/Pakistan and the Middle East, northern China and the east coast of Australia and New Zealand. The overall spatial distribution of the grid cells with a high variation hardly changes over time, but the maximum variation as well as the geographic extent of the uncertain areas increases after 2030 (e.g., into the west of the USA and further north in western Russia). In 2100, this development reverses, most probably due to the more limited number of models reporting values for that time step.

Areas of uncertainty of forest dynamics can be found in all major forest areas globally, including boreal, temperate and tropical forests. Hotspots of variation are mainly located at the edges between forested and nonforested areas, rather than in the center of large forested areas (e.g., in the high latitudes of Siberia). While this pattern emerges already in 2030, it becomes more obvious in 2050 and 2100.

For pasture, recognizable variations are present in almost every grid cell containing pastures, although the amount of variation is low compared with cropland and forest. Hotspots can be hardly detected in 2030, while in 2050 central Brazil, central India and western Australia emerge as the regions with the highest variation. In 2100, further parts of North and South America, the Sahara surrounding area and large parts of East Asia are increasingly uncertain, although still below the uncertainty found in cropland change projections.

### Quantity and allocation disagreement in pairwise map comparisons

**Figure 6:**
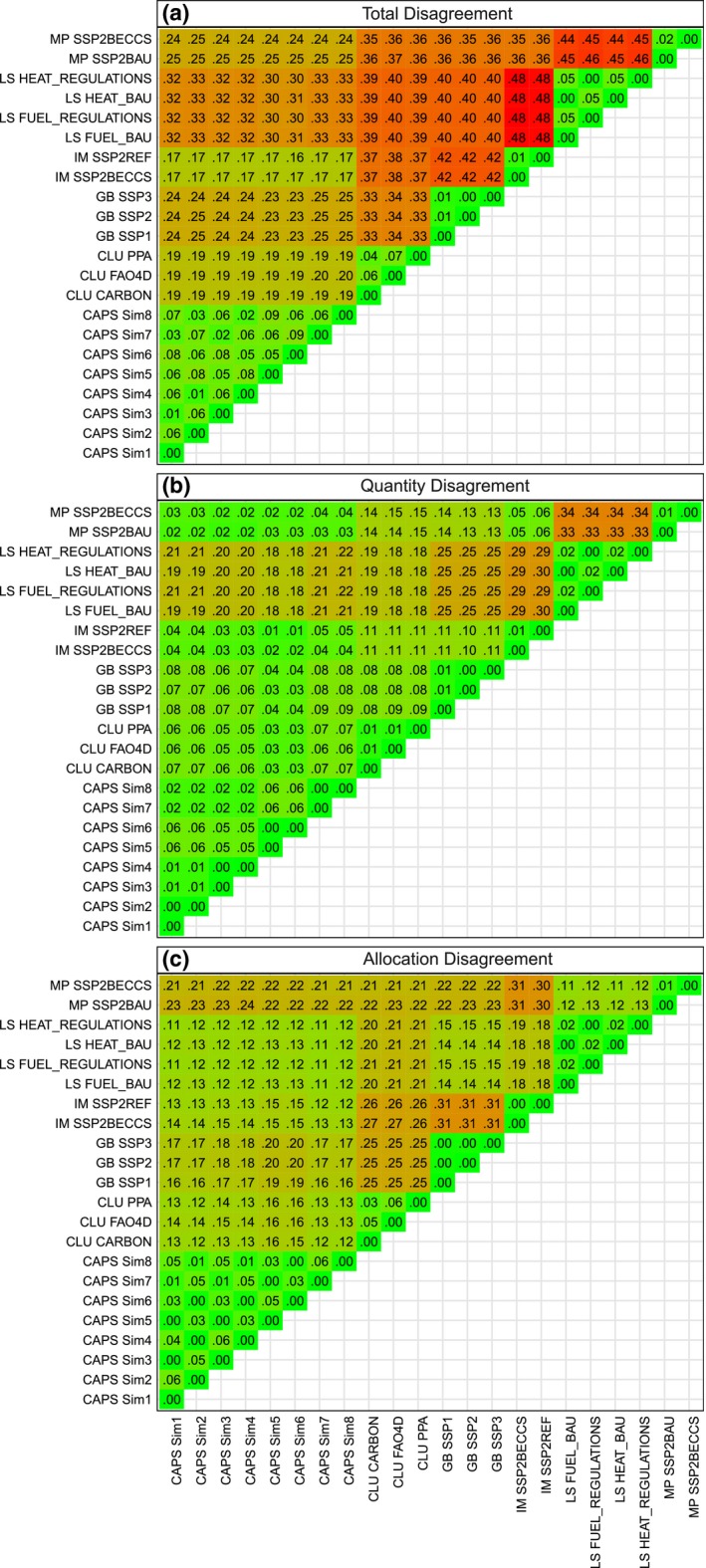
Decomposition of disagreement components for each pairwise comparison in 2030. (a) Total disagreement at 0.5 × 0.5 degree grid cell level, (b) quantity disagreement component (= total disagreement when whole globe considered as one pixel) and (c) allocation disagreement component (= difference of the former two components). The numbers represent the fraction of global land area. CLU = CLUMondo, GB = GLOBIOM, IM = IMAGE, LS = LandSHIFT, MP = MAgPIE, for scenario decoding see Table S5.

The total disagreement is generally low between different scenarios of the same model at the 0.5 × 0.5 degree resolution in 2030, while differences between models are higher (Fig. 6a). We found maximum values of 6% and 7% within‐model disagreement for the CLUMondo mitigation scenarios compared with the reference scenario and up to 9% for several scenarios of the CAPS model, respectively. Consistently, all within‐model disagreements are lower than the smallest disagreement between scenarios from any two different models (minimum value of 16% between IMAGE SSP2 reference and CAPS Sim6 scenarios).

Maximum disagreements between models can be found between IMAGE and LandSHIFT results, where on 48% of the total land area (excluding Antarctica and Greenland) there is no agreement about the LULC categories. LandSHIFT corresponds least with any of the other models, which is mostly due to different quantities of the various LULC types (~70% of the total disagreement, Fig. 6a, b), likely a result of the different scenarios considered by this model. Comparisons between maps of any model with the CAPS model resulted in the smallest disagreements, which can most probably be ascribed to the limited amount of categories compared in these cases (cropland, pasture and other natural, Table 1). CLUMondo scenarios yield between 33% and 38% total disagreement when compared to scenarios of GLOBIOM, IMAGE and MAgPIE, where comparison with GLOBIOM gained the highest similarity and with IMAGE the lowest. The allocation component of the total disagreement is thereby larger than the quantity disagreement throughout. Maps of the GLOBIOM, IMAGE and MAgPIE models show similar amounts of total disagreement, ranging from 35% (MAgPIE and GLOBIOM or IMAGE, respectively) to 42% (IMAGE and GLOBIOM). However, while IMAGE and MAgPIE are almost consistent in terms of global quantities (quantity disagreement between 5% and 6%), their disagreement with GLOBIOM is both due to quantity and allocation.

### Confusion of LULC types across scenarios

**Figure 7:**
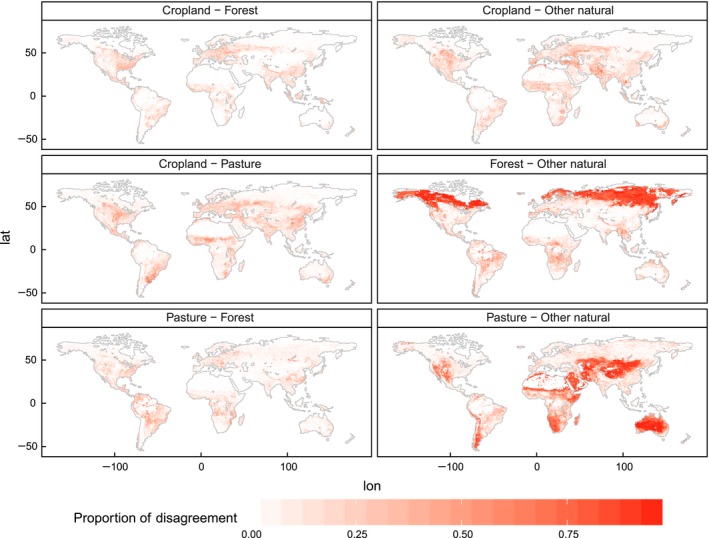
Land type confusion on grid cell level in 2030. The grid cell values represent the proportion of each confusion type on total disagreement per grid cell (urban not shown due to the low confusion rates). Only grid cells where total disagreement is >10% are considered.

Figure 7 displays the average confusion (i.e., maps show different LULC types in the same grid cell at the same time) of LULC types in the maps of all possible pairwise comparisons, which we show as an illustration for the year 2030. Values represent the proportion of a particular confusion type (e.g., cropland in one map and forest in another, Fig. 7 top left) on the total disagreement in a grid cell. We removed confusions with urban (very small amount of grid cells and portions) and grid cells with a total average disagreement lower than 10 % for reasons of clarity.

Most of the disagreement between models can be assigned to the ambiguity between pasture and other natural land in large parts of the world, with hotspots in Australia, Central Asia, large parts of the African continent outside of tropical forests, the southern part of South America and also the central and western part of North America. In the high latitudes, the disagreement between forest and other natural land is the dominating confusion type. This pattern, however, only appears in grid cells with smaller amounts of total disagreement (<25%, Fig. S8). Compared with that, all other confusion types are low, although other confusions of LULC types also contribute substantially to the total disagreement.

## Discussion

### Hotspots of uncertainty

The comparison of model results in this paper has been made both for LULC changes and for the actual LULC areas. Differences between the actual areas and the simulated changes have different origins and different impacts on the assessment of uncertainty in spatially explicit LULC projections (Brown *et al*., 2013). Important components determining differences in assessment of changes vs. the actual areas relate to the impact of input data on the projections and the spatial allocation of global or regional LULC change at regular grid level. Both issues are, however, related because the models usually allocate changes in relation to the LULC representation at a former time step (e.g., agricultural land expands at the edges of already cultivated land), which makes the influence of input data even more important. Input data have been indicated as a major uncertainty source in future LULC change trajectories before (Smith *et al*., 2010; Fritz *et al*., 2011; Verburg *et al*., 2011; Popp *et al*., 2014b), which is confirmed by our results; especially for pasture and forest, initial variation dominates the uncertainty in the scenarios under consideration, but also cropland shows substantial deviations in the start values of the models. This can be attributed to different sources of input data used to initialize the models, which rely on variant definitions and data acquisition approaches. Moreover, while models simulating aggregate change at global or regional levels are often based on statistical data, the initial areas of spatial land change models are often derived from available land‐cover maps based on remote sensing data or harmonized products. What is actually defined as a forest is, for example, highly dependent on the origin and framework observational data originates in. Sexton *et al*. (2015) recently reported large differences (up to 13% of the earth's land area) between global satellite based forest data products concluding that the main reason for this discrepancy originates in definition issues rather than the technological limitation of earth observation sensors and the algorithms applied to derive land‐cover and land‐use categories [although this also still remains an uncertainty factor, e.g., Friedl *et al*. (2010)]. These kinds of data in turn are implemented to different extents in the models of our comparison either directly (e.g., Bontemps *et al*. (2011) in LandSHIFT; Hansen *et al*. (2003) in CLUMondo) or indirectly by compiled products of census and remote sensing data or potential natural vegetation maps from DGVMs (e.g., Erb *et al*. (2007) in MAgPIE).

Sexton *et al*. (2015) further identified high disagreements in the considered forest data products at the transition zones of boreal forest to tundra and (sub)tropical forest to savannah biomes; areas which we could also detect as highly variable in our model and scenario dataset. Therefore, it seems highly likely that these discrepancies in observational data propagate into model outputs and this is further confirmed by the dominance of initial conditions in the variance decomposition. Although the importance of these initial aspects strongly decreases when only considering LULC changes (i.e., removing the differences in the initial conditions), the geographic pattern remains very similar, which may be, to some extent, attributed to the impact of different input data. Nevertheless, the transitions between different biomes are also areas where many of the LULC models allocate change as result of the gradient of environmental conditions or through the implementation of climate change in the allocation mechanisms that would affect the suitability of these zones for different LULC types. It is therefore these zones that gather multiple uncertainties in the LULC modeling process that call for more attention for studying these areas to help reduce the uncertainty in projections for these areas.

To reduce uncertainty in initial LULC data, recently a number of initiatives have been taken by data assimilation or crowdsourcing strategies (Fritz *et al*., 2012; Tuanmu & Jetz, 2014). We expect feeding models with consensus LULC products as initial data will certainly reduce the differences in model outcome and facilitate further model comparisons, concentrating on structural model uncertainty. However, such harmonization strategies will also obscure the uncertainty embedded in the current state of land use and land cover and would only be justified by an actual reduction of the uncertainty of the data.

While the data input and definition issue mainly dominate the uncertainties in projections of forest and pasture, the analysis of LULC changes also shows wide variation across the models and scenarios in most of the regions for cropland projections. These results indicate that, even if a proper depiction of the current state of LULC existed, uncertainty in future LULC related to the model structure and scenario assumptions remain. Part of this variation can be explained by the scenarios used in our comparison, whose input assumptions were not harmonized. Different scenarios are expected to result in a variation in LULC outcomes and are a common way of addressing uncertainties in major socioeconomic developments or evaluating the sensitivity of land use to policy alternatives. However, the partitioning of the variation clearly shows that only a part of the variation can be explained by the differences in scenarios and that, often, the results of different scenarios of the same model are more similar to the results of the same scenario by different models.

Several hotspots of uncertainty in the gridded maps are located in areas characterized by rapid past LULC changes (Lepers *et al*., 2005). Thus, several areas of special interest for future LULC change trajectories represent also areas of high uncertainties in current LULC modeling. Integration of assessments on local or regional scales may help to improve the representation of LULC changes in global‐scale applications.

### Scaling issues in uncertainty assessment

The analysis of land‐cover and land‐use changes further revealed a scale dependency in the uncertainty patterns. The results at the grid level suggest that the actual hotspots of uncertainty follow the borders of globally important biomes rather than administrative borders of geographically or economically delimited world regions. Therefore, the uncertainty in certain regions depicted in regional‐level uncertainty maps may only apply to specific parts of such a region and should be interpreted with care.

All considered LULC types show this pattern to a certain extent, while it is most obvious in the forest category. Two of the uncertainty hotspots for cropland can be found, for example, at a north to south gradient in the center of the North American continent and in the southwest of Russia, both rendering the whole regions uncertain at the regional level in 2030 and 2050 (Figs 2 and 5). Another example is the above‐mentioned transition zone between boreal forest and tundra ecosystems.

Uncertainty assessment at the scale of large world regions is not capable of revealing the actual hotspots of LULC uncertainty. First, the average uncertainty in a world region could be misleading as it removes the heterogeneity of uncertainty patterns within the regions. Second, actual hotspots located at the boundaries of two or more administrative units could dilute the importance of the hotspot by dividing the disagreement between the regions which individually are not being identified as a hotspot.

Thus, the level of spatial detail in analyzing uncertainty matters and should be carefully considered, especially in applications utilizing LULC change models at different spatial resolutions. Ideally, uncertainty assessments should account for a variety of spatial scales and alternative regional subdivisions to narrow down the areas of substantial uncertainties as our study has demonstrated. This would allow to investigate the impact of different spatial resolutions on the uncertainty in LULC trajectories in more detail and may suggest alternative regional subdivision for future model development.

### Implications for environmental assessments

The output of LULC change models is widely utilized in global‐ and regional‐scale environmental assessments. Too often land‐use reconstructions or projections are regarded as observations without accounting for uncertainty while our results show that these projections contain serious sources of uncertainty. In the Climate Model Intercomparison Project (CMIP) simulations for the Intergovernmental Panel on Climate Change (IPCC) harmonized historical and future LULC change trajectories are used (Taylor *et al*., 2012). The future LULC change trajectories for the four RCPs are provided by four different IAMs and smoothly connected to the HYDE historical LULC reconstruction (Hurtt *et al*., 2011; Klein Goldewijk *et al*., 2011). Our results indicate that this strategy is likely to have consequences for downstream model input data for two reasons. First, although harmonization ensures common starting conditions for different models, it obscures the uncertainty about the current state of LULC that strongly propagates in model results. Second, in the current approach of simulating the RCPs, the influence of model diversity on LULC change trajectories is not considered as each scenario is simulated by a different model. Both initial data and model parameters have been shown to contribute substantially to the uncertainty in LULC projections, hampering a good comparison of the impact of scenario conditions on the final outcomes. Thus, further sensitivity exercises addressing the uncertainty in LULC for the same scenario in climate impact models are required to test the sensitivity of the outputs and quantify the uncertainty. The strong spatial patterns in the uncertainty suggest that also the downstream impacts of the uncertainties in impact assessment are spatially diverse. The correspondence of regions with high uncertainty to regions that may have important impacts on climate change suggests the importance of focusing on further uncovering the sources of uncertainty in these regions to avoid error propagation in environmental assessments.

### Limitations

Unlike previous intercomparison exercises (Popp *et al*., 2014b; Schmitz *et al*., 2014), we did not make any effort to either harmonize the participating simulations to common scenario constraints or to calibrate models to a common starting map. This was done to ensure a wider participation of models and integrate LULC change models from different domains that are normally not part of the intercomparison exercises that are strongly related to the IPCC process. However, this approach makes comparison more challenging, in particular the interpretation of results. The diversity of scenario assumptions applied in the models and the scenario parameterization approach adds a certain extent of uncertainty to the model results in our database which is independent of model structure and cannot be quantified adequately. We, thus, do not propose that uncertainty can be completely reduced by model improvement. As LULC is driven by individual human decisions, future LULC is uncertain by nature. However, the results of the variance decomposition at regional level and pairwise comparisons at gridded level indicate that model structure and allocation schemes are an important source of uncertainty and need further attention at various scales. The article also does not aim to evaluate or judge individual model performance as, inherent to the chosen approach of comparison in which initial data and scenarios are not harmonized, this is not possible. Rather, we have identified areas of high uncertainty and different sources of uncertainty related to this. Applying these models to gain further knowledge about the socioeconomic and environmental challenges of the future requires a good understanding of the range of modeling approaches available and awareness about uncertainty sources. With our approach, we were able to identify hotspots of uncertainty in regional and spatially explicit LULC change modeling, thereby suggesting locations where further research should focus on to improve global‐scale trajectories of LULC change.

## Supporting information


**Figure S1.** Aggregated world regions as applied in the regional analysis.
**Figure S2.** Schematic overview of the analysis conducted in this study.
**Figure S3.** Variation of land use changes for 43 scenarios of 11 models.
**Figure S4.** Projections of land cover areas [Mha] for cropland, pasture and forest.
**Figure S5.** Full results of the variance decomposition for land type cropland.
**Figure S6.** Full results of the variance decomposition for land type pasture.
**Figure S7.** Full results of the variance decomposition for land type forest.
**Figure S8.** Land type confusion on grid cell level in 2030 (grid cells with more than 25% total disagreement).
**Table S1.** Regional aggregation of the different models to 12 common world regions.
**Table S2.** Adjustment factors per land type and model.
**Table S3.** Default variables for socio‐economic variables used in the scenario parameterization.
**Table S4.** Example of the cross‐tabulation matrix approach for pairwise map comparisons.
**Table S5.** Parameterization of models and scenarios for multiple regressions and analysis of variance.
