# Supplementary material for: Hotspots of uncertainty in land‐use and land‐cover change projections: a global‐scale model comparison
Source: Glob Chang Biol. 2016 Jun 8;22(12):3967–83. doi: 10.1111/gcb.13337 (PMC5111780; doi:10.1111/gcb.13337)
Supplement: Supplementary file 1 — Figure S1. Aggregated world regions as applied in the regional analysis. Figure S2. Schematic overview of the analysis conducted in this study. Figure S3. Variation of land use changes for 43 scenarios of 11 models. Figure S4. Projections of land cover areas [Mha] for cropland, pasture and forest. Figure S5. Full results of the variance decomposition for land type cropland. Figure S6. Full results of the variance decomposition for land type pasture. Figure S7. Full results of the variance decomposition for land type forest. Figure S8. Land type confusion on grid cell level in 2030 (grid cells with more than 25% total disagreement). Table S1. Regional aggregation of the different models to 12 common world regions. Table S2. Adjustment factors per land type and model. Table S3. Default variables for socio‐economic variables used in the scenario parameterization. Table S4. Example of the cross‐tabulation matrix approach for pairwise map comparisons. Table S5. Parameterization of models and scenarios for multiple regressions and analysis of variance. [file GCB-22-3967-s001.docx]

# Supporting Information: Hotspots of LULC change uncertainty

**Preprocessing of model results for regional level analysis**

**Spatial aggregation**

Areas for cropland, pasture and forest were extracted from the model outputs for 12 aggregated world regions (Figure S1), which coincide with the regions of the FARM model except for India, which was merged with the remaining South Asian countries.

| 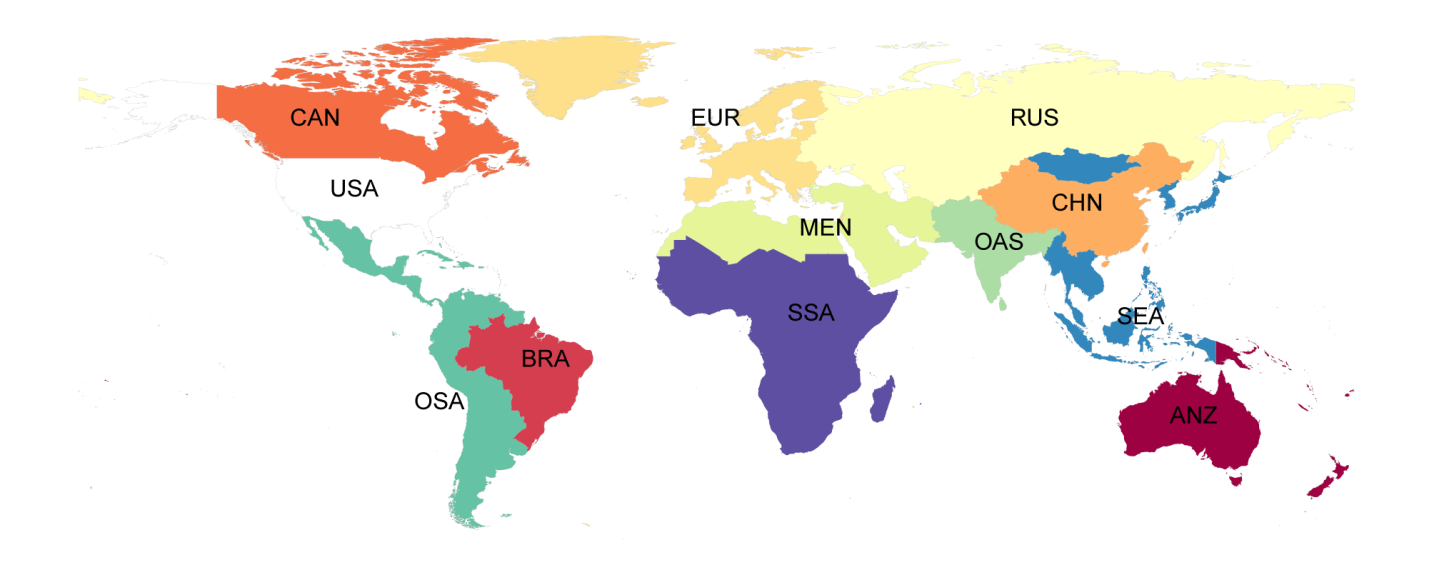 |
| --- |
| Figure S1 Aggregated world regions as applied in the regional analysis; **ANZ** Australia, New Zealand and Oceania, **BRA** Brazil, **CAN** Canada, **CHN** China including Taiwan, Macau and Hong Kong, **EUR** Europe, **MEN** Middle East and North Africa including Turkey, **OAS** South Asia including India, **OSA** South and Middle America excluding Brazil, **RUS** Russia including eastern Europe, **SEA** Southeast and East Asia including Mongolia, **SSA** Sub-Saharan Africa, **USA** United States of America |

We used the ISO3 codes to relate individual countries to world regions in the 5 regional level models and aggregated the regions for every model as shown in Table S1. A review of the ISO3 codes for every model revealed that models sometimes do not agree about the assignment of individual countries to the same aggregated world region (e.g. Mongolia is part of China in GCAM and MAGNET, while it is part of Southeast Asia in FARM and AIM). We therefore adjusted the LULC areas for some aggregated world regions and models as following:

1. Derivation of the correct mapping of model regions to the aggregated world regions (Table S1).
2. Extraction of all countries that are not accordingly mapped to the same aggregated world regions in all models from ISO3 code assignments.
3. Assigning the correct mapping for every country according to the majority of models (so we could keep the introduced uncertainty as low as possible the less outputs we process).
4. For all models which disagree with this majority mapping, we calculated the proportion of the respective countries in the 2010 areas per LULC type based on FAOSTAT (2015), reduced the area for the aggregated region by this factor and increased the area of the correct world region.

A detailed overview, which regions were adjusted for every model is shown in Table S2. This procedure assumes that (1) the values for 2010 LULC type areas reported by FAOSTAT (2015) agree with the areas reported by the individual models and (2) the contribution to the land type areas of a country in 2010 remains constant until the end of the simulation period. Although this will introduce another dimension of uncertainty into the comparison results (that cannot be quantified, since the real country level areas per model are not known), it reduces the obvious errors that would originate in an incorrect mapping of LULC type areas to the aggregated regions.

We further aggregated the outputs of the six spatially explicit models as following:

1. construction of 12 polygons covering the aggregated world regions from the ISO3 data base
2. multiplication of the fractional value per 0.5 x 0.5  degree grid cell with the area of the grid cell (using the *area()* function of the R raster package (Hijmans, 2015))
3. calculation of zonal sum for every region

| Table S1 Aggregation of the different model regions to 12 common world regions; naming according to model documentation and ISO3 country codes | | | | | |
| --- | --- | --- | --- | --- | --- |
| Acronym | Aggregated world region | AIM regions | MAGNET regions | GCAM regions | PLUM regions |
| ANZ | Australia/New Zealand | XOC | Oceania | Australia_NZ | AUS, NZL |
| BRA | Brazil | BRA | Brazil | BRA | BRA |
| CAN | Canada | CAN | Canada | CAN | CAN |
| CHN | China | CHN | ChinaPlus | CHN | CHN, TWN |
| EUR | Europe | XER, XE25 | EU16, EU12, REaEurope, RWeEurope | EU-15, EU-12, Europe_Non_EU, European FreeTrade Association | ALB, AUT, BEL, BGR, BIH, FRAU, CHE, CYP, CZE, DEU, DNK, ESP, EST, FIN, FRA, GBR, GRC, HRV, HUN, IRL, ITA, LTU, LUX, LVA, MKD, MLT, NLD, NOR, POL, PRT, ROU, SVK, SVN, SWE |
| RUS | Russia/Central Asia | CIS | RussiaPlus, UkrainePlus, AsiaStan | RUS, Europe_Eastern, Central Asia | ARM, AZE, BLR, GEO, KAZ, KGZ, MDA, RUS, TJK, TKM, UKR, UZB |
| MEN | Middle East/North Africa | XME, XNF, TUR | MiddleEast, NoAfrica, Turkey | Middle East, Africa_Northern | ARE, DZA, EGY, IRN, ISR, JOR, KWT, LBN, LBY, MAR, MRT, MAR, SAU, SYR, TUN, TUR, YEM |
| OAS | India/South Asia | XSA, IND | IndiaPlus | IND, PAK, South Asia | BGD, IND, LKA, MDV, NPL, PAK |
| OSA | South/Central America | XLM | Mexico, RestCeAmerica, RestSoAmerica | ARG, COL, MEX, Central America and Carribean, South America_Southern, SouthAmerica_Northern | ARG, BHS, BLZ, BOL, BRB, CHL, COL, CRI, CUB, DOM, ECU, GTM, GUY, HND, HTI, JAM, MEX, NIC, PAN, PER, PRY, SLV, SUR, TTO, URY, VEN |
| SEA | Southeast Asia | XSE, JPN | Japan, Korea, IndonesiaPlus, SaEaAsia | IDN, JPN, KOR, Southeast Asia | BRN, FJI, IDN, JPN, KHM, KOR, LAO, MMR, MNG, MYS, NCL, PHL, PRK, SLB, THA, VNM, VUT |
| SSA | Sub-Saharan Africa | XAF | SoAfrica, EaAfrica, WeAfrica | ZAF, Africa_Southern, Africa_Eastern, Africa_Western | AGO, BDI, BEN, BFA, BWA, CAF, CIV, CMR, COG, COM, CPV, DJI, ETH, GAB, GHA, GIN, GMB, GNB, KEN, LBR, LSO, MDG, MLI, MOZ, MUS, MWI, NAM, NER, NGA, RWA, SDN, SEN, SLE, SWZ, TCD, TGO, TZA, UGA, ZAF, ZMB, ZWE |
| USA | United States of America | USA | USA | USA | USA |

Table S2 Adjustment factor per land type and region for the AIM, GCAM and MAGNET areas; numbers represent percentage of the 2010 area derived from FAOSTAT (2015) for every aggregated region; only countries with land area > 1 Mha mentioned.

| Region | Cropland | | | Pasture | | | Forest | | | Countries | | |
| --- | --- | --- | --- | --- | --- | --- | --- | --- | --- | --- | --- | --- |
|  | AIM | GCAM | MAGNET | AIM | GCAM | MAGNET | AIM | GCAM | MAGNET | AIM | GCAM | MAGNET |
| ANZ | + 3.2 | + 3.9 | + 7.3 | + 0.2 | + 0.2 | + 0.3 | + 22.6 | + 237.7 | - | FJI, NCL, PNG, SLB, VUT | FJI, NCL, PNG, SLB, VUT | FJI, NCL, PNG, SLB, VUT |
| BRA | - | - | - | - | - | - | - | - | - | - | - | - |
| CAN | - | - | - | - | - | - | - | - | - | - | - | - |
| CHN | + 0.6 | - | - 1.0 | - | - | - | - | - | - | TWN | - | - |
| EUR | + 0.2 | - 16.3 | - | + 0.5 | - 18.3 | - 1.7 | - | - 4.8 | - | ISR | - | ISR |
| RUS | - | - 0.3 | - | - | - 24.2 | - | - | - 1.2 | - | - | - | - |
| MEN | - 0.4 | + 40.3 | - 1.3 | + 1.5 | + 5.5 | - 0.2 | + 2.2 | + 1120.5 | - | ESH | TUR | - |
| OAS | - 0.8 | - | - | - 0.1 | - | - | - 54.2 | - | - | - | - | - |
| OSA | + 0.1 | + 0.1 | + 1.0 | - | + 0.3 | + 0.7 | - | + 0.1 | - | - | FLK | GUY |
| SEA | - 0.8 | - 1.0 | - 1.8 | - | + 827.1 | + 1288.7 | + 0.2 | - 7.7 | - | - | MNG | MNG |
| SSA | - | - | - | - 0.6 | - | - | - 0.1 | - | - | - | - | - |
| USA | - | - | - |  | - | - 0.1 |  | - 0.2 | - | - | - | - |

**Rescaling to 2010**

The starting years of the models are different (Table 1), while 2010 is the earliest year which is covered by all models (except for CAPS, which was linearly interpolated between 2005 and 2030 values on the basis of the 12 world regions). However, the LULC areas in 2010 reported by the individual modes and scenarios also vary due to historical definition issues and uncertainty in land statistics. To allow comparisons without the effect of this initial variation, we calculated the LULC changes projected by the individual models as proportion of the 2010 LULC areas for each of the 12 regions. Areas at each decadal end year between 2010 and 2100 were divided by the respective 2010 area for each LULC type. Missing values were linearly interpolated.

**Preprocessing of model results for gridded analysis at 0.5 x 0.5 degree**

Gridded models came with a range of different map projections, spatial resolutions and legends. All model results were preprocessed to a common format. We decided to use fractional grid cell area at a 0.5 x 0.5 degree regular grid in an un-projected WGS84 coordinate system, since this ensured the lowest impact on original model outputs and could be achieved by spatial aggregation. The range of thematic detail (ranging from 3 classes for CAPS to 30 classes for LandSHIFT) was resolved by mapping all legends to a common legend of five broader LULC types: cropland, pasture, forest, urban and other natural. If less than the five classes were available the remaining classes were assumed to be merged with the other natural category. Individual model results were treated as following:

**CAPS** Maps were provided in the coordinate system as used in the analysis. The legend only covered cropland and pastures. An *other natural* category was calculated as [1- (cropland + pasture)]. Forest and urban land cover was therefore assumed to be merged with the *other natural* class. As CAPS model outputs were not available for the year 2010, which was the reference year for our analysis, we assumed the LULC areas at 2005 (the starting map of CAPS) to be the same in 2010.

**CLUMondo** Model output was submitted for the years 2000 to 2040 annually. The maps for every individual year have a spatial resolution of 9.25 x 9.25 km (~5arcminute at the equator) in an equal area map projection (WORLD ECKERT IV) based on WGS84 datum. Every grid cell was classified in a dominant way according to the land system legend described in van Asselen and Verburg (2012). Land systems are amongst others characterized by a certain composition of fractional membership per grid cell to five land use types, namely cropland, pasture, forest, urban and bare land. These properties vary across 24 world regions and were additionally provided by the CLUMondo team. We used this lookup tables to reclassify the land system maps for every year to maps of fractions per grid cell for every above mentioned LULC class. Subsequently the maps were projected to 5 arcminute WGS84 datum and aggregated to 0.5 x 0.5 degree applying an area weighted average.

**GLOBIOM** Maps were provided in 5 arcminute resolution (WGS84 datum) and aggregated to 0.5 x 0.5 degree resolution applying an area weighted average. The legend does not include an *urban* class. Urban was therefore assumed to be merged with the *other natural* class.

**IMAGE** Maps were provided in the coordinate system and legend as used in the analysis. No preprocessing was therefore applied.

**LandSHIFT** Maps were submitted in 5 arcminute resolution (WGS84 datum) using a dominant classification based on the GlobCover legend (Bontemps *et al.*, 2011) with some additional classes. Reclassification of the legend was done using the dominant functional type in each category as recommended by the LandSHIFT team to the classes cropland, pasture, forest, built-up and other natural. Subsequently all maps were aggregated to 0.5 x 0.5 degree resolution applying an area weighted average.

**MAgPIE** Maps were provided in coordinate system and legend as used in the analysis. No harmonization was therefore applied.

**Methodological details**

*
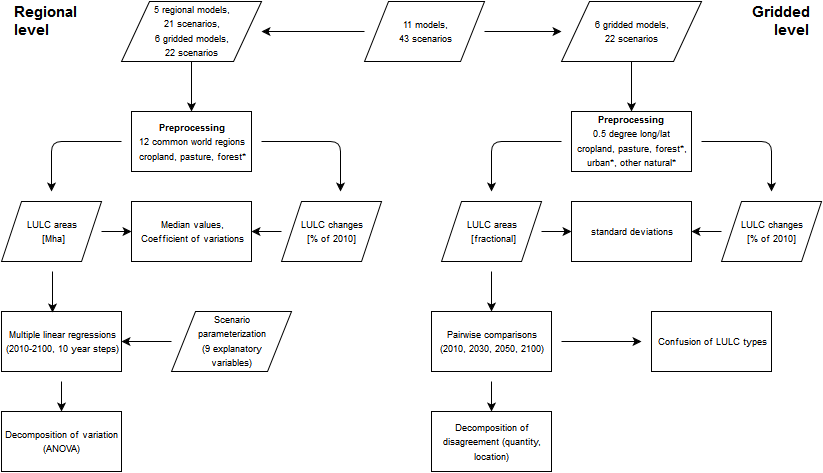
*

Figure S2 Schematic overview of analysis conducted in this study. Parallelograms represent data (both original and processed), while rectangles represent steps of analysis. *LULC categories were not available for all models.

**Scenario parameterization**

Regression analysis followed by an ANOVA was used to decompose the variability in modeled LULC areas at each decadal end year of the simulation period. Explanatory variables as shown in Table 2 were used to describe the individual scenarios. Default values available for SSP/RCP and SRES (IIASA, 2015, Nakicenovic & Swart, 2000, van Vuuren & Carter, 2014, van Vuuren *et al.*, 2011) were used, while deviating numbers were used if available for the individual scenarios (Table S3). Inequality ratio was calculated as ratio of per capita incomes between OECD and non-OECD countries. Climate scenarios could have different meanings in the individual model projections (e.g. climate impact on yield projections or biofuel implementation to reach mitigation goals). CO_2_ concentration in 2100 was therefore used as a proxy to describe climate change within the scenarios.

Table S3 Default variables for socio-economic variables used in the scenario parameterization (IIASA, 2015, Nakicenovic & Swart, 2000, van Vuuren & Carter, 2014, van Vuuren *et al.*, 2011). Table adopted from Alexander *et al.* (in review).

| Scenario | Global population 2100 (billion) | GDP total growth rate 2100 (annual %) | Inequality 2100 ratio | CO_2_ 2100 (ppm CO_2_e) | Technology change | Global trade |
| --- | --- | --- | --- | --- | --- | --- |
| A1 (A1B) | 7 | 2.90 | 1.61 | 700 | Rapid | High |
| A2 | 15 | 2.30 | 4.18 | 850 | Slow | Constrained |
| B1 | 7 | 2.50 | 1.81 | 550 | Medium | High |
| B2 | 10 | 2.20 | 3.02 | 620 | Medium | Constrained |
| SSP1 | 6.9 | 2.44 | 1.35 | 650 (RCP 4.5) | Rapid | Moderate |
| SSP2 | 9.0 | 2.38 | 1.69 | 850 (RCP 6.0) | Medium | Moderate |
| SSP3 | 12.7 | 1.64 | 3.47 | 1370 (RCP 9.5) | Slow | Constrained |
| SSP4 | 9.4 | 1.91 | 3.78 | 850 (RCP 6.0) | Medium | Moderate |
| SSP5 | 7.4 | 3.11 | 1.26 | 1370 (RCP 8.5) | Rapid | High |
| Present | 6.9 | 0 | 5.80 | 390 (Present) | None | Moderate |

**Map comparison**

Pontius and Cheuk (2006) propose a cross-tabulation approach to compare any two soft-classified maps. We adapted this approach to compare the results (= maps of LULC fractions at the grid cell level) of the six gridded LULC models in years 2010, 2030, 2050 and 2100. All unique model and scenario combinations were therefore pairwise compared (CAPS Sim1 compared to CAPS Sim2, CAPS Sim1 compared to CAPS Sim3, …, MAgPIE SSP2BECCS compared to MAgPIE SSP2BAU), resulting in 253 individual comparisons for 2010 and 2030, 190 for 2050 and 120 for 2100.

The cross-tabulation matrix is calculated as following: Firstly, the agreement per legend item in each valid grid cell of the two maps under consideration is calculated as the minimum of the two fractional values (step1). In a second step, disagreements for both maps are calculated as the difference between the actual fraction in the original maps and the agreement map calculated in step 1. Subsequently, the disagreement per LULC type in one map is distributed among the LULC categories in the second map proportional to the disagreements of this map resulting in a cross-tabulation matrix (Table S4).

The cross-tabulation matrix was calculated at three levels, each yielding different kind of information:

1. 0.5 x 0.5 degree grid cell level: This calculation yields the information about the disagreement and agreement of LULC types in one grid cell. The matrix entries (Table S4) represent fractions of one grid cell in this case. The information at grid cell level was used to calculate the average confusion, i.e. the fraction of the grid cell which is a particular LULC type in one map and a different LULC type in the other map, across all pairwise comparisons as shown in Figure 7.
2. Mean value of all 0.5 x 0.5 grid cells. The matrix entries (Table S4) represent fractions of global land area in this case. The summary matrix was used to calculate total agreement between the maps as shown in Figure 6.
3. Calculated considering the whole globe as one grid cell. This equals the differences per LULC type in global quantities, which is referred to as quantity disagreement in this article, since it does not consider how the LULC areas allocated on the regular grid. Thus, this represents the maximum agreement between the two maps.

Since the individual grid cells represent different shares of global land area in the longitude/latitude grid, an area weighting was applied whenever grid cells were aggregated to a coarser resolution. For mathematical proof and detailed explanation of the method see Pontius and Cheuk (2006).

Table S4 Example of the cross-tabulation matrix following Pontius and Cheuk (2006) (**c:** cropland, **p:** pasture, **f:** forest, **u:** urban, **o**: other natural). Shading distinguishes the interpretation of the entries: dark grey cells represent percentage of study area, where both maps agree about LULC type. Medium grey shading (off-diagonal) represents the percentage of study area, where both maps do not agree about LULC type. Light grey shading (marginal row and column) represents the total share of each LULC type in the respective map. The sum of diagonal entries is the total agreement between the two maps. This matrix was calculated for each pairwise comparison at the grid cell level as well as summarized over the whole globe.

|  | | Map 1 | | | | |  |
| --- | --- | --- | --- | --- | --- | --- | --- |
|  |  | i = c | i = p | i = f | i = u | i = o | Total map 2 |
| Map 2 | j = c | f_cc_ | f_pc_ | f_fc_ | f_uc_ | f_oc_ | $\sum f_{ic}$ |
|  | j = p | f_cp_ | f_pp_ | f_fp_ | f_up_ | f_op_ | $\sum f_{ip}$ |
|  | j = f | f_cf_ | f_pf_ | f_ff_ | f_uf_ | f_of_ | $\sum f_{if}$ |
|  | j = u | f_cu_ | f_pu_ | f_fu_ | f_uu_ | f_ou_ | $\sum f_{iu}$ |
|  | j = o | f_co_ | f_po_ | f_fo_ | f_uo_ | f_oo_ | $\sum f_{io}$ |
|  | Total map 1 | $\sum f_{cj}$ | $\sum f_{pj}$ | $\sum f_{fj}$ | $\sum f_{uj}$ | $\sum f_{oj}$ | $\sum f_{ij}$  $if i=j$ |

Table S5 Parameterization of models and scenarios for multiple regression and analysis of variance; table adapted from Alexander *et al.* (in review) and supplemented by additional scenarios; **CGE** Computable general equilibrium model, **PE** Partial equilibrium model, **Hybrid** Allocation model using demand from CGE or PE

| Model | Scenario ID | Socio-economic scenario | Climate scenario | Global population 2100 (Billions) | GDP growth to 2100 (%/year) | Inequality ratio 2100 | CO_2_ 2100 (ppm) | Technology change | Global trade | Model Type | Number model cells (log) |
| --- | --- | --- | --- | --- | --- | --- | --- | --- | --- | --- | --- |
| AIM | SSP1 | SSP1 | Present | 6.9 | 2.4 | 1.35 | 390 | Rapid | Moderate | CGE | 1.2 |
| AIM | SSP2 | SSP2 | Present | 9 | 2.4 | 1.69 | 390 | Medium | Moderate | CGE | 1.2 |
| AIM | SSP3 | SSP3 | Present | 12.7 | 1.6 | 3.47 | 390 | Slow | Constrained | CGE | 1.2 |
| CAPS | Sim1 | SSP3 | RCP 8.5 | 12.7 | 1.6 | 3.47 | 1370 | Slow | Constrained | Hybrid | 5.4 |
| CAPS | Sim2 | SSP3 | RCP 8.5 | 12.7 | 1.6 | 3.47 | 1370 | Slow | Constrained | Hybrid | 5.4 |
| CAPS | Sim3 | SSP5 | RCP 8.5 | 7.4 | 3.1 | 1.26 | 1370 | Rapid | High | Hybrid | 5.4 |
| CAPS | Sim4 | SSP5 | RCP 8.5 | 7.4 | 3.1 | 1.26 | 1370 | Rapid | High | Hybrid | 5.4 |
| CAPS | Sim5 | B1 | RCP 4.5 | 7 | 2.5 | 1.81 | 650 | Medium | High | Hybrid | 5.4 |
| CAPS | Sim6 | B1 | RCP 4.5 | 7 | 2.5 | 1.81 | 650 | Medium | High | Hybrid | 5.4 |
| CAPS | Sim7 | A2 | RCP 8.5 | 15 | 2.3 | 4.18 | 1370 | Slow | Constrained | Hybrid | 5.4 |
| CAPS | Sim8 | A2 | RCP 8.5 | 15 | 2.3 | 4.18 | 1370 | Slow | Constrained | Hybrid | 5.4 |
| CLUMondo | FAO4D | SSP2 | Present | 9 | 2.4 | 1.69 | 390 | Medium | Moderate | Hybrid | 6.7 |
| CLUMondo | CARBON | SSP2 | Present | 9 | 2.4 | 1.69 | 390 | Medium | Moderate | Hybrid | 6.7 |
| CLUMondo | PPA | SSP2 | Present | 9 | 2.4 | 1.69 | 390 | Medium | Moderate | Hybrid | 6.7 |
| FARM | SSP1 RCP 4.5 | SSP1 | RCP 4.5 | 6.9 | 2.4 | 1.35 | 650 | Rapid | Moderate | CGE | 1.1 |
| FARM | SSP1 present climate | SSP1 | Present | 6.9 | 2.4 | 1.35 | 390 | Rapid | Moderate | CGE | 1.1 |
| FARM | SSP2 RCP 6.0 | SSP2 | RCP 6.0 | 9 | 2.4 | 1.69 | 850 | Medium | Moderate | CGE | 1.1 |
| FARM | SSP2 present climate | SSP2 | Present | 9 | 2.4 | 1.69 | 390 | Medium | Moderate | CGE | 1.1 |
| FARM | SSP3 RCP 8.5 | SSP3 | RCP 8.5 | 12.7 | 1.6 | 3.47 | 1370 | Slow | Constrained | CGE | 1.1 |
| FARM | SSP3 present climate | SSP3 | Present | 12.7 | 1.6 | 3.47 | 390 | Slow | Constrained | CGE | 1.1 |
| GCAM | SSP1 | SSP1 | Present | 6.9 | 2.4 | 1.35 | 390 | Rapid | Moderate | PE | 1.5 |
| GCAM | SSP2 | SSP2 | Present | 9 | 2.4 | 1.69 | 390 | Medium | Moderate | PE | 1.5 |
| GCAM | SSP3 | SSP3 | Present | 12.7 | 1.6 | 3.47 | 390 | Slow | Constrained | PE | 1.5 |
| GCAM | SSP4 | SSP4 | Present | 9.4 | 1.9 | 3.78 | 390 | Medium | Moderate | PE | 1.5 |
| GCAM | SSP5 | SSP5 | Present | 7.4 | 3.1 | 1.26 | 390 | Rapid | High | PE | 1.5 |
| GLOBIOM | SSP1 | SSP1 | Present | 6.9 | 2.4 | 1.35 | 390 | Rapid | Moderate | PE | 6.9 |
| GLOBIOM | SSP2 | SSP2 | Present | 9 | 2.4 | 1.69 | 390 | Medium | Moderate | PE | 6.9 |
| GLOBIOM | SSP3 | SSP3 | Present | 12.7 | 1.6 | 3.47 | 390 | Slow | Constrained | PE | 6.9 |
| IMAGE | SSP2_450_BECCS | SSP2 | RCP 2.6 | 9 | 2.4 | 1.69 | 490 | Medium | Moderate | Hybrid | 5.4 |
| IMAGE | SSP2_450_REF | SSP2 | RCP 2.6 | 9 | 2.4 | 1.69 | 490 | Medium | Moderate | Hybrid | 5.4 |
| LandSHIFT | FUEL_BAU | SSP2 | Present | 9 | 2.4 | 1.69 | 390 | Medium | Moderate | Rule-based | 6.9 |
| LandSHIFT | FUEL_REGULATIONS | SSP2 | Present | 9 | 2.4 | 1.69 | 390 | Medium | Moderate | Rule-based | 6.9 |
| LandSHIFT | HEAT_BAU | SSP2 | Present | 9 | 2.4 | 1.69 | 390 | Medium | Moderate | Rule-based | 6.9 |
| LandSHIFT | HEAT_REGULATIONS | SSP2 | Present | 9 | 2.4 | 1.69 | 390 | Medium | Moderate | Rule-based | 6.9 |
| MAGNET | SSP1 | SSP1 | RCP 4.5 | 6.9 | 2.4 | 1.35 | 650 | Rapid | Moderate | CGE | 1.4 |
| MAGNET | SSP2 | SSP2 | RCP 6.0 | 9 | 2.4 | 1.69 | 850 | Medium | Moderate | CGE | 1.4 |
| MAGNET | SSP3 | SSP3 | RCP 8.5 | 12.7 | 1.6 | 3.47 | 1370 | Slow | Constrained | CGE | 1.4 |
| MAgPIE | BAU | SSP2 | Present | 9 | 2.4 | 1.69 | 390 | Medium | Moderate | PE | 5.4 |
| MAgPIE | BECCS | SSP2 | Present | 9 | 2.4 | 1.69 | 390 | Medium | Moderate | PE | 5.4 |
| PLUM | A1 | A1 | A1 | 7 | 2.9 | 1.61 | 700 | Rapid | High | Rule-based | 2.2 |
| PLUM | A2 | A2 | A2 | 15 | 2.3 | 4.18 | 850 | Slow | Constrained | Rule-based | 2.2 |
| PLUM | B1 | B1 | B1 | 7 | 2.5 | 1.81 | 550 | Medium | High | Rule-based | 2.2 |
| PLUM | B2 | B2 | B2 | 10 | 2.2 | 3.02 | 620 | Medium | Low | Rule-based | 2.2 |

**Additional results**

**
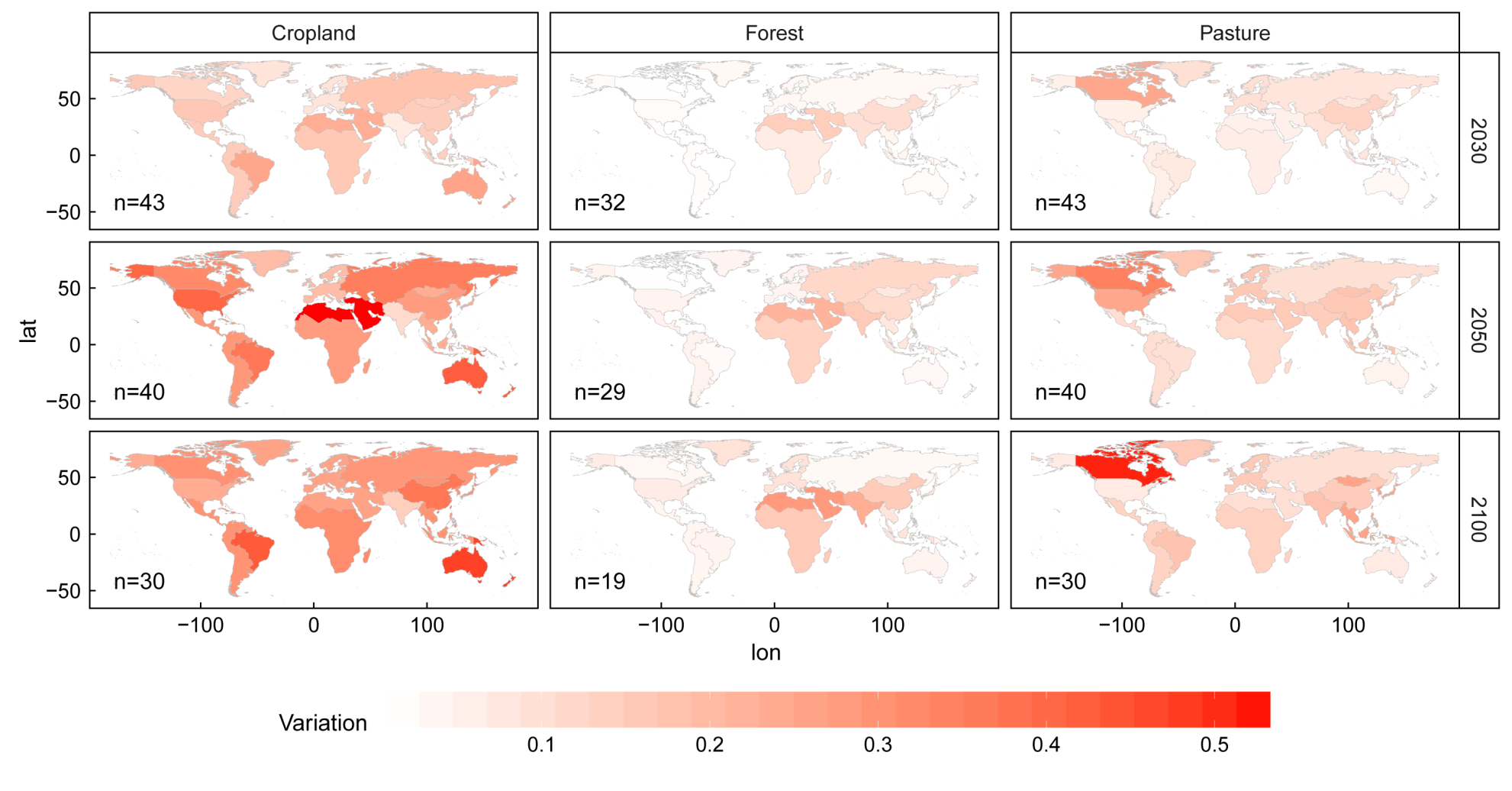
**

Figure S3 Variation of land use changes for 43 scenarios of 11 models in cropland, forest and pasture category; variation expressed as coefficient of variation; n depicts the number of models underlying the calculation of COV.


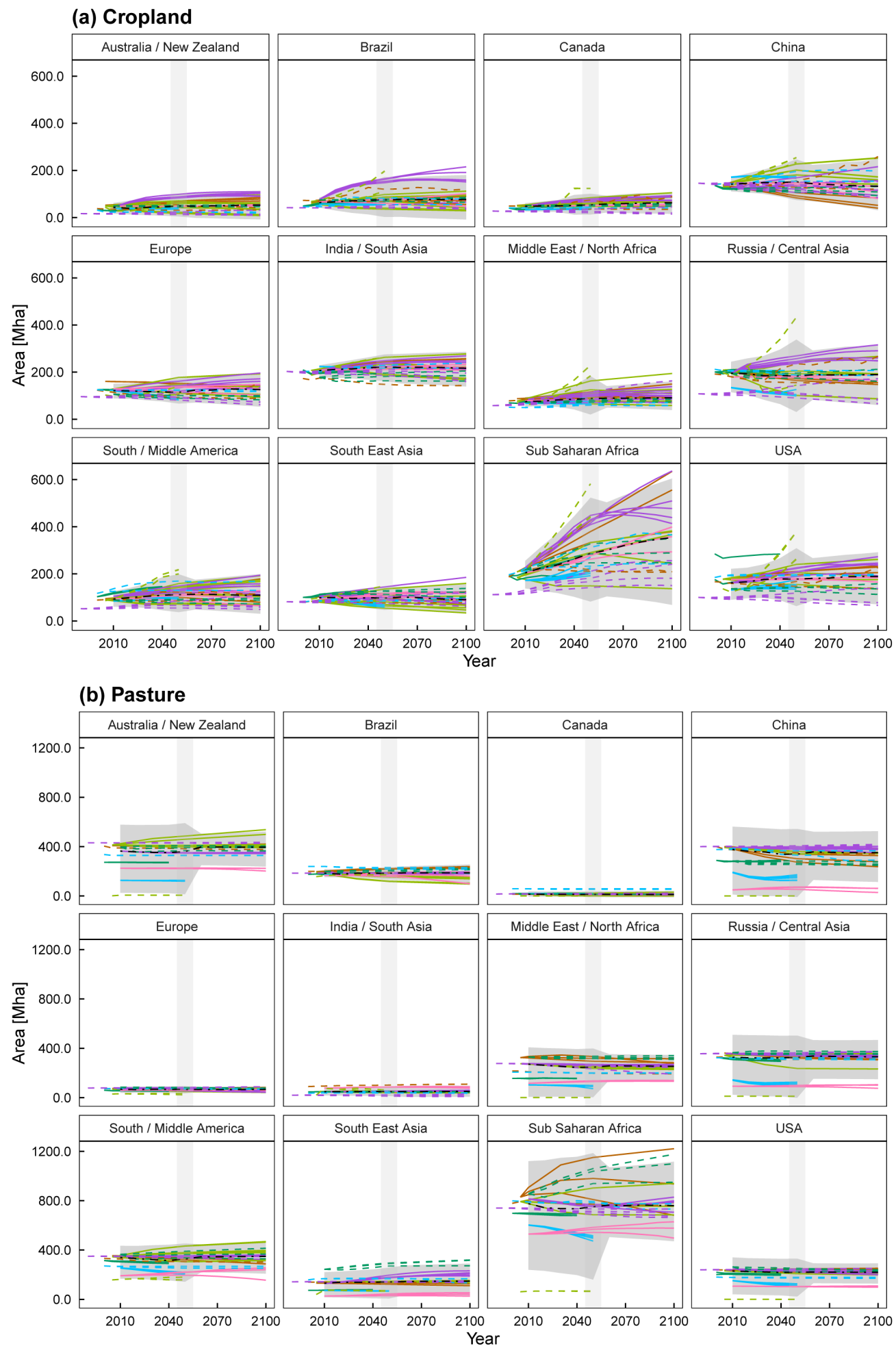


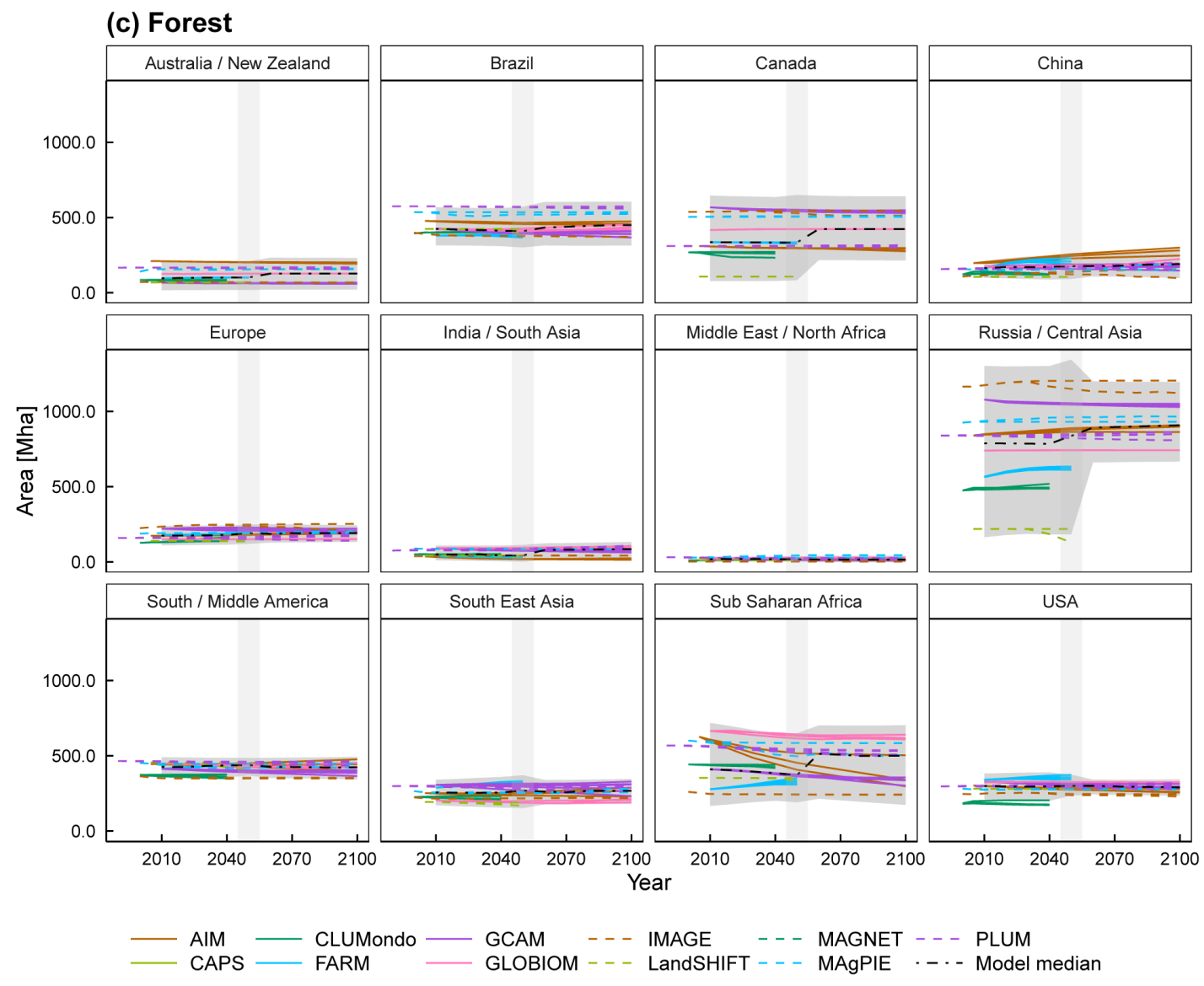


Figure S4 Projections of land cover areas [Mha] for (a) cropland, (b) pasture and (c) forest of 11 models for a total of 43 scenarios. The grey shading represents the 95 % interval of model results, while the vertical grey bar indicates a change in the amount of models between 2040 and 2060. Note the different ranges of scales applied for cropland, pasture and forest categories.

**Variance decomposition – full results**


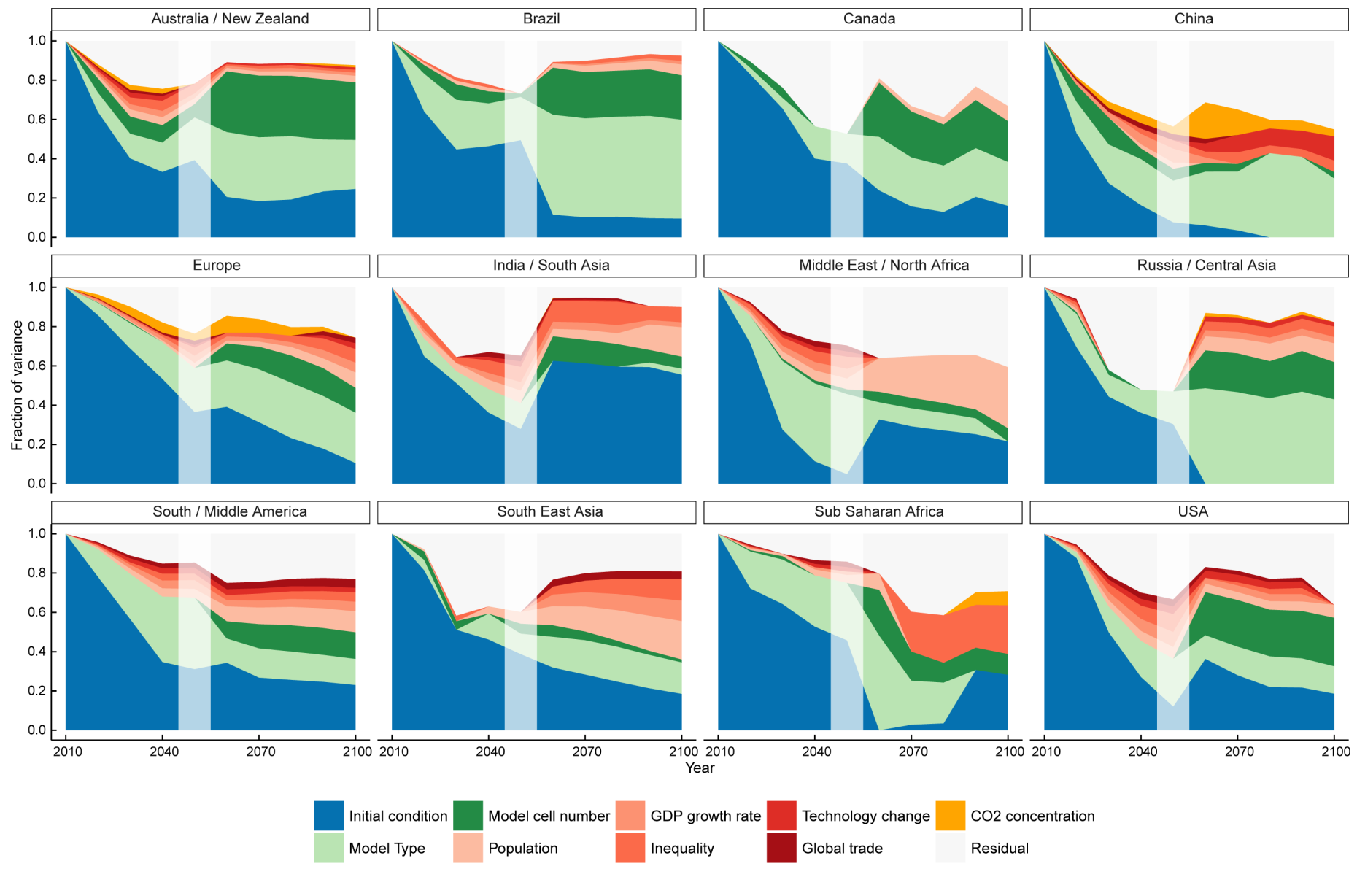


Figure S5 Full results of the variance decomposition for land type cropland.


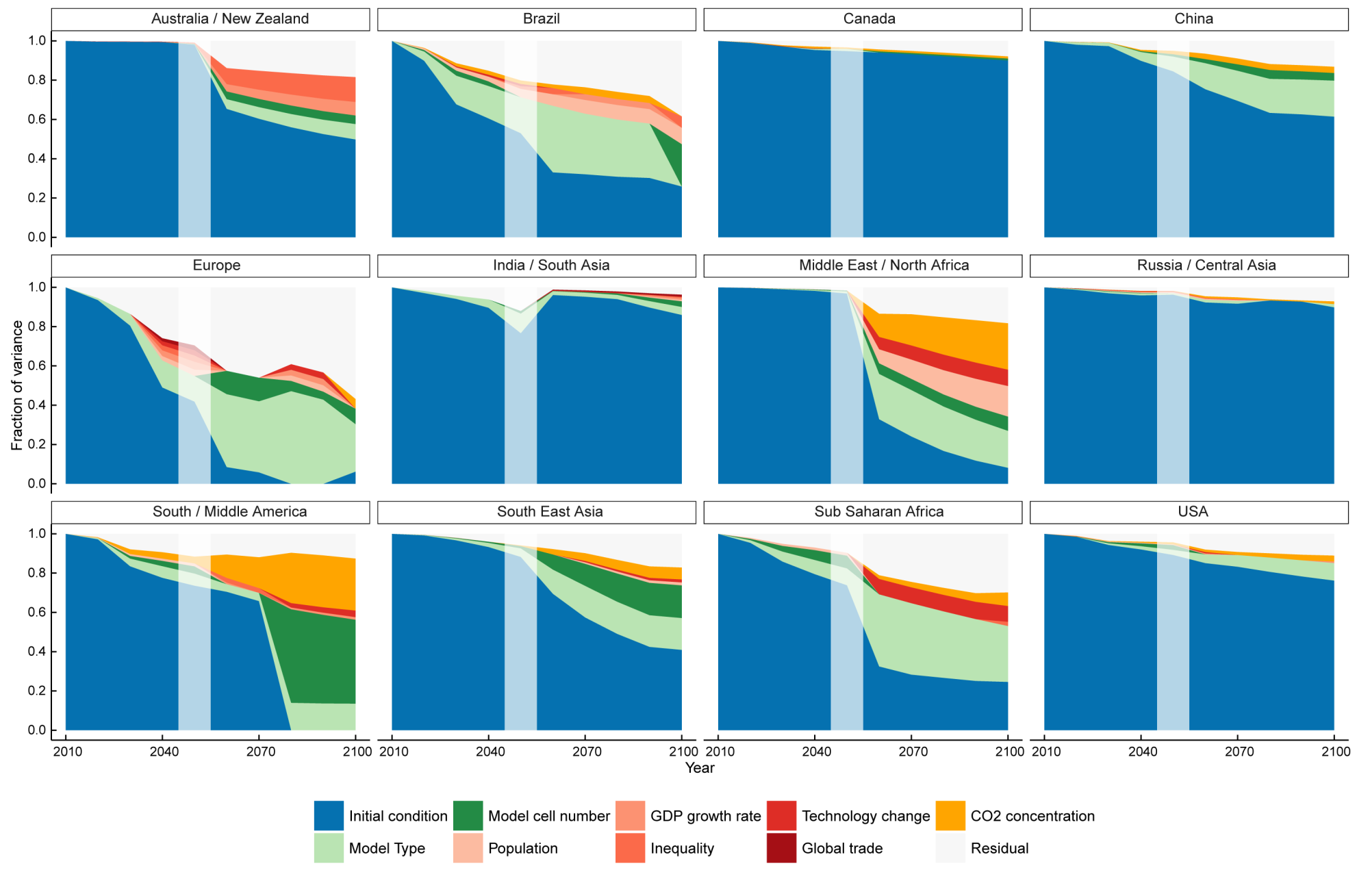


Figure S6 Full results of the variance decomposition for land type pasture.


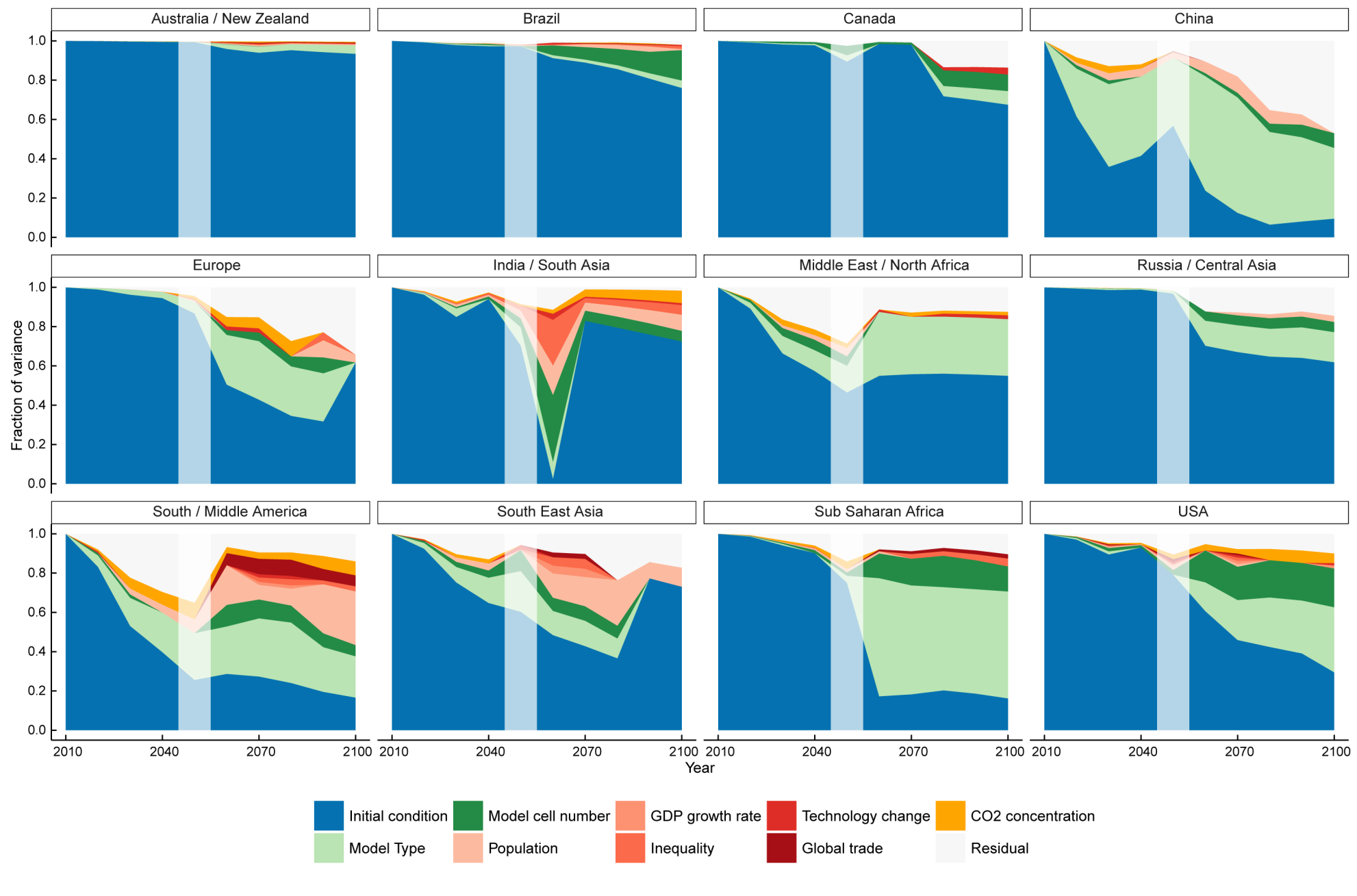


Figure S7 Full results of the variance decomposition for land type forest.


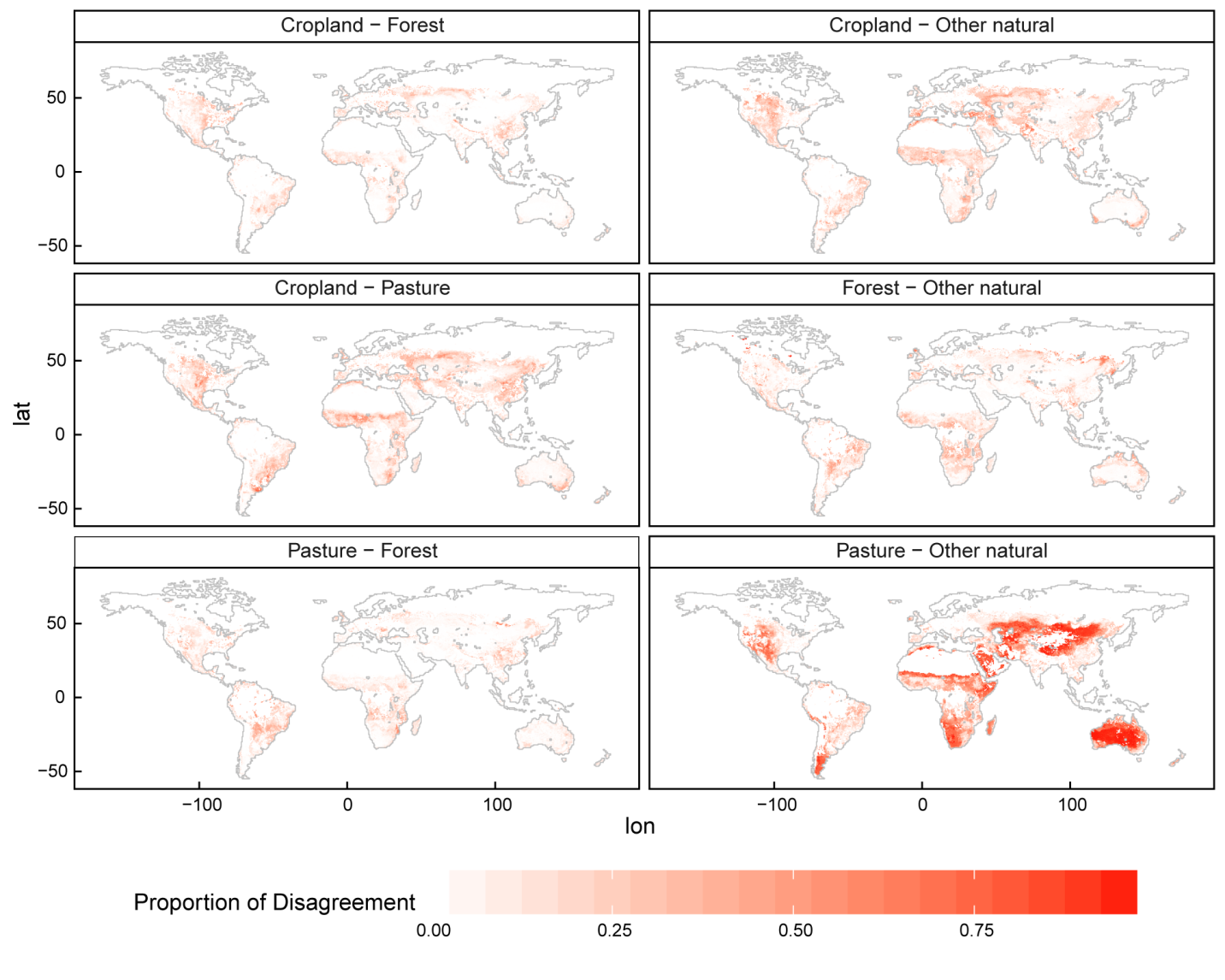


Figure S8 Land type confusion on grid cell level in 2030. The grid cell values represent the proportion of each confusion type on total disagreement per grid cell (built-up not shown due to the low confusion rates). Only grid cells where total disagreement is greater than 25% are considered

# References

Alexander P, Prestele R, Verburg P *et al.* (in review) Assessing uncertainties in land cover projections.

Bontemps S, Defourny P, Van Bogaert E, Arino O, Kalogirou V, Perez JR (2011) GLOBCOVER 2009 - Products Description and Validation Report.

FAOSTAT (2015) Resources/Land. Food and Agriculture Organization of the United Nations, Rome, Italy.

Hijmans R.J. (2015) raster: Geographic Data Analysis and Modeling. R package version 2.5-2. https://CRAN.R-project.org/package=raster

IIASA (2015) SSP Database (version 0.93), International Institute for Applied System Analysis, Laxenburg, Austria.

Nakicenovic N, Swart R (eds) (2000) *Special report on emission scenarios: a special report of working group III of the intergovernmental panel on climate change,* Cambridge, IPCC.

Pontius RG, Cheuk ML (2006) A generalized cross‐tabulation matrix to compare soft‐classified maps at multiple resolutions. International Journal of Geographical Information Science*,* **20**, 1-30.

Van Asselen S, Verburg PH (2012) A Land System representation for global assessments and land-use modeling. Global Change Biology*,* **18**, 3125-3148.

Van Vuuren DP, Carter TR (2014) Climate and socio-economic scenarios for climate change research and assessment: reconciling the new with the old. Climatic Change*,* **122**, 415-429.

Van Vuuren DP, Edmonds J, Kainuma M *et al.* (2011) The representative concentration pathways: an overview. Climatic Change*,* **109**, 5-31.
